# Local and long-range atomic/magnetic structure of non-stoichiometric spinel iron oxide nanocrystallites

**DOI:** 10.1107/S2052252520013585

**Published:** 2021-01-01

**Authors:** Henrik L. Andersen, Benjamin A. Frandsen, Haraldur P. Gunnlaugsson, Mads R. V. Jørgensen, Simon J. L. Billinge, Kirsten M. Ø. Jensen, Mogens Christensen

**Affiliations:** a Department of Chemistry and Interdisciplinary Nanoscience Center, Aarhus University, Langelandsgade 140, Aarhus C, DK-8000, Denmark; b Department of Physics and Astronomy, Brigham Young University, N283 ESC, Provo, Utah 84602, USA; c Science Institute, University of Iceland, Dunhaga 5, Reykjavík, 107, Iceland; d MAX IV Laboratory, Lund University, PO Box 118, Lund, SE-221 00, Sweden; e Department of Applied Physics and Applied Mathematics, Columbia University, 500 W. 120th Street, New York 10027, USA; f Condensed Matter Physics and Materials Science Department, Brookhaven National Laboratory, PO Box 5000, Upton, New York 11973, USA; g Department of Chemistry and Nanoscience Center, University of Copenhagen, Universitetsparken 5, København Ø, DK-2100, Denmark

**Keywords:** supercritical hydro­thermal synthesis, magnetite (Fe_3_O_4_), maghemite (γ-Fe_2_O_3_), magnetic nanoparticles, synchrotron powder X-ray diffraction, neutron total scattering, magnetic pair distribution function

## Abstract

Spinel iron oxide nanoparticles of different mean sizes in the range 10–25 nm have been prepared by surfactant-free up-scalable near- and super-critical hydro­thermal synthesis pathways and characterized using a wide range of advanced structural characterization methods to provide a highly detailed structural description. The atomic structure is examined by combined Rietveld analysis of synchrotron powder X-ray diffraction (PXRD) data and time-of-flight neutron powder-diffraction (NPD) data. The local atomic ordering is further analysed by pair distribution function (PDF) analysis of both X-ray and neutron total-scattering data. It is observed that a non-stoichiometric structural model based on a tetragonal γ-Fe_2_O_3_ phase with vacancy ordering in the structure (space group *P*4_3_2_1_2) yields the best fit to the PXRD and total-scattering data. Detailed peak-profile analysis reveals a shorter coherence length for the superstructure, which may be attributed to the vacancy-ordered domains being smaller than the size of the crystallites and/or the presence of anti-phase boundaries, faulting or other disorder effects. The intermediate stoichiometry between that of γ-Fe_2_O_3_ and Fe_3_O_4_ is confirmed by refinement of the Fe/O stoichiometry in the scattering data and quantitative analysis of Mössbauer spectra. The structural characterization is complemented by nano/micro-structural analysis using transmission electron microscopy (TEM), elemental mapping using scanning TEM, energy-dispersive X-ray spectroscopy and the measurement of macroscopic magnetic properties using vibrating sample magnetometry. Notably, no evidence is found of a Fe_3_O_4_/γ-Fe_2_O_3_ core-shell nanostructure being present, which had previously been suggested for non-stoichiometric spinel iron oxide nanoparticles. Finally, the study is concluded using the magnetic PDF (mPDF) method to model the neutron total-scattering data and determine the local magnetic ordering and magnetic domain sizes in the iron oxide nanoparticles. The mPDF data analysis reveals ferrimagnetic collinear ordering of the spins in the structure and the magnetic domain sizes to be ∼60–70% of the total nanoparticle sizes. The present study is the first in which mPDF analysis has been applied to magnetic nanoparticles, establishing a successful precedent for future studies of magnetic nanoparticles using this technique.

## Abstract 2

The atomic, magnetic and nano-structure of hydro­thermally synthesized non-stoichiometric spinel iron oxide nanoparticles are examined in detail using a wide range of advanced structural characterization methods. The present study constitutes the first use of the recently developed magnetic pair-distribution-function analysis method to model the local magnetic structure in nanoparticles.

## 1. Introduction   

In recent years, magnetic nanoparticles of the spinel iron oxide compounds, *i.e.* magnetite (Fe_3_O_4_) and maghemite (γ-Fe_2_O_3_), have attracted immense interest, in particular, owing to their applications in the biomedical field, *e.g.* as functionalized medicine carriers for drug delivery (Pankhurst *et al.*, 2003 ▸; Berry & Curtis, 2003 ▸), as contrast agents in magnetic resonance imaging scans (Lee *et al.*, 2007 ▸; Na *et al.*, 2009 ▸) and in magnetic hyperthermia cancer treatment (Quinto *et al.*, 2015 ▸; Jang *et al.*, 2009 ▸). For these purposes, magnetic spinel iron oxide nanoparticles are generally favoured over other nanocrystalline magnetic compounds owing to their biocompatibility, low price, good magnetic properties and high resistance to corrosion (Lu *et al.*, 2007 ▸; Valenzuela, 2012 ▸). Numerous studies report synthetic pathways for obtaining iron oxide nanoparticles with specific sizes, shapes and properties (Hyeon *et al.*, 2001 ▸; Park *et al.*, 2007 ▸; Kovalenko *et al.*, 2007 ▸; Andrés Vergés *et al.*, 2008 ▸; Kim *et al.*, 2009 ▸; Guardia *et al.*, 2012 ▸, 2014 ▸; Wetterskog *et al.*, 2013 ▸; Mirabello *et al.*, 2016 ▸; Qiao *et al.*, 2017 ▸; Feld *et al.*, 2019 ▸; Muro-Cruces *et al.*, 2019 ▸), but despite the substantial research interest, the atomic and magnetic structures of spinel iron oxide nanoparticles synthesized by even the most common methods in materials chemistry (including the hydro­thermal method studied here) are still unclear (Bremholm *et al.*, 2009 ▸; Adschiri *et al.*, 1992 ▸, 2000 ▸; Park, 2009 ▸). As the magnetic properties of the spinel iron oxide phases (and magnetic materials in general) are determined by the complex interplay between their crystal-, magnetic and nano-structures, a reliable structural characterization is essential for understanding their macroscopic magnetic behaviour and for rationally designing synthesis pathways for preparation of nanoparticles with specific properties.

**Figure 1:**
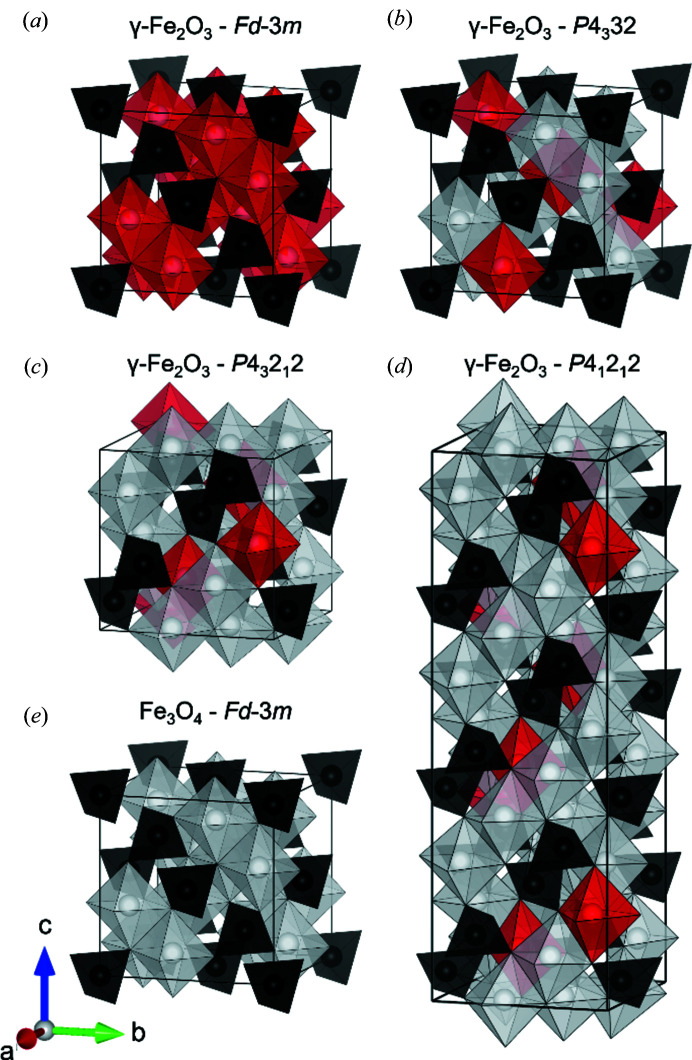
(*a*)–(*d*) Illustrations of the possible γ-Fe_2_O_3_ structural configurations and (*e*) the structure of Fe_3_O_4_. The black and white polyhedra represent tetrahedral [FeO_4_] and octahedral [FeO_6_] units, respectively. The red polyhedra highlight the partially occupied or unoccupied octahedral sites, illustrating the degree of vacancy ordering. The illustrations have been made using the structure-visualization software *VESTA* (Momma & Izumi, 2011 ▸).

The Fe_3_O_4_ and γ-Fe_2_O_3_ compounds adopt very similar atomic structures, as they both crystallize in the spinel structure, which features tetrahedrally and octahedrally coordinated metal sites. However, while Fe_3_O_4_ contains both trivalent Fe^3+^ and divalent Fe^2+^ ionic species, all iron in γ-Fe_2_O_3_ is in the trivalent state. Charge neutrality in the γ-Fe_2_O_3_ spinel structure is maintained by the introduction of vacancies on octahedrally coordinated cation sites. In the bulk, ordering of these vacancies is known to occur and has been subject to extensive research. As a result, the structure of γ-Fe_2_O_3_ has over recent decades been reported in several different space groups as illustrated in Figs. 1 ▸(*a*)–1 ▸(*d*).

The possible structural configurations include the following. (*a*) The simple magnetite-like cubic spinel in space group 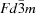 with vacancies randomly distributed on the octahedral site (deBoer & Dekkers, 1996 ▸). (*b*) A closely related cubic structure in space group *P*4_3_32 with two distinct octahedral sites and vacancies on only one of them (Braun, 1952 ▸; Shmakov *et al.*, 1995 ▸). (*c*) A tetragonal structure in space group *P*4_3_2_1_2 with three distinct octahedral sites and vacancies on only one of them (Greaves, 1983 ▸) (see Table 1 ▸ for more details). (*d*) A superstructure of the *P*4_3_2_1_2 cell in space group *P*4_1_2_1_2 with *c/a* = 3 and further vacancy ordering (Oosterhout & Rooijmans, 1958 ▸; Shmakov *et al.*, 1995 ▸; Jørgensen *et al.*, 2007 ▸; Somogyváari *et al.*, 2002 ▸).

**Figure 2:**
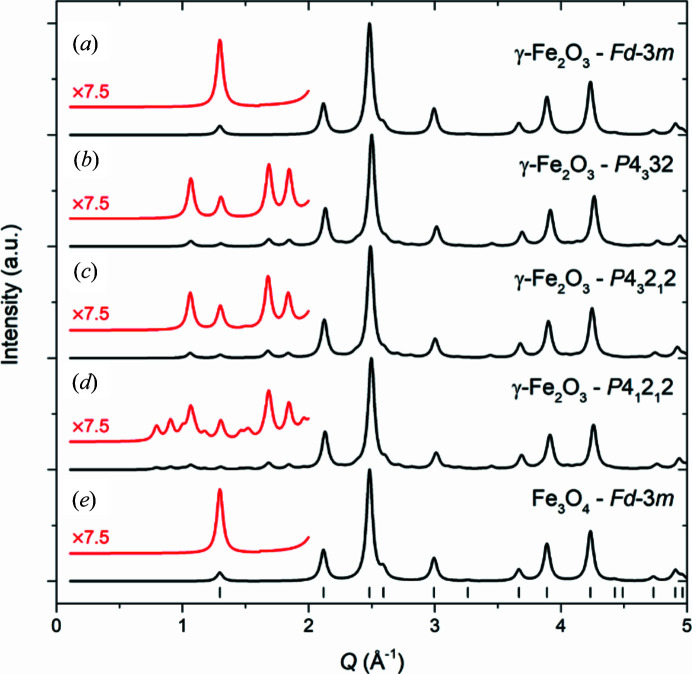
Simulated PXRD patterns for 15 nm nanocrystallites of γ-Fe_2_O_3_ (space groups: 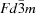, *P*4_3_32, *P*4_3_2_1_2 and *P*4_1_2_1_2) and Fe_3_O_4_ (space group: 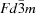). The additional superstructure reflections in the low *Q* region arising from the ordering of vacancies in the structure are magnified and illustrated in red.

Nanosized spinel iron oxide particles are, in the literature, generally assumed to be either of the stoichiometric Fe_3_O_4_ or γ-Fe_2_O_3_ in the archetypical 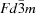 spinel space group. However, conclusive structural characterization is rarely provided, precluding reliable discrimination between the magnetite and the vacancy-ordered/disordered maghemite structures. Fig. 2 ▸ shows simulated powder X-ray diffraction (PXRD) patterns of nanocrystallites with the above-mentioned structures of γ-Fe_2_O_3_ and Fe_3_O_4_. In all cases, the main diffraction peaks can be indexed by the simple spinel structure. However, the lower symmetry caused by the ordering of vacancies in the γ-Fe_2_O_3_ unit cell gives rise to very weak superstructure reflections. As highlighted in Fig. 2 ▸, the vacancy ordering in the *P*4_3_32 and *P*4_3_2_1_2 structures yields three additional characteristic peaks in the low *Q* region (*Q* = momentum transfer), while the tripled unit cell in *P*4_1_2_1_2 further increases the number of Bragg reflections. Previous studies of spinel iron oxide nanoparticle systems have indicated that the average iron oxidation state, the degree of vacancy ordering and the magnetic properties depend on the synthesis method, reaction conditions and nanoparticle size (Jørgensen *et al.*, 2007 ▸; Frison *et al.*, 2013 ▸; Salazar *et al.*, 2011 ▸; Cooper *et al.*, 2020 ▸; Bastow *et al.*, 2009 ▸; Grau-Crespo *et al.*, 2010 ▸; Somogyváari *et al.*, 2002 ▸).

The hydro­thermal method is among the most widespread synthesis pathways used for the preparation of nanosized spinel iron oxide particles. Interestingly, the nanocrystalline products obtained from the hydro­thermal treatment of ammonium iron(III) citrate precursors are generally reported to be Fe_3_O_4_ in the literature (Bremholm *et al.*, 2009 ▸; Adschiri *et al.*, 1992 ▸, 2000 ▸; Park, 2009 ▸). However, since the ammonium iron(III) citrate precursor only contains iron in the ferrous state (Fe^III^), the hydro­thermal treatment would intuitively be expected to lead to the formation of purely trivalent α-Fe_2_O_3_ or γ-Fe_2_O_3_. In order to rationalize the formation of the mixed valence Fe_3_O_4_ from the purely Fe(III)-containing precursor, it has been hypothesized that the citrate ligand molecules decompose to yield carbon monoxide during the hydro­thermal treatment. The formed CO gas, which mixes with the hydro­thermal media, can then partially reduce Fe^3+^ in the precursor to Fe^2+^, while itself being oxidized to CO_2_, allowing formation of Fe_3_O_4_ (Adschiri *et al.*, 1992 ▸; Bremholm *et al.*, 2009 ▸).

It is well established that the formed iron oxide nanocrystallites adopt the spinel structure, however, the exact nature of the atomic ordering and the average iron-oxidation state in the product have not been unambiguously determined. Conventional Rietveld refinement of PXRD data in theory allows determination of the elemental stoichiometry of the compounds from the refined site-occupation fractions, however, these parameters are among the least well behaved in structural modelling (Blake & Clegg, 2009 ▸). In addition, the combination of the almost identical X-ray scattering powers of the Fe^2+^ and Fe^3+^ ions, the size broadening of the Bragg peaks owing to the reduced crystallite sizes and the high backgrounds owing to Fe fluorescence at the Cu *K*α X-ray energy conventionally used for structural investigations, causes the site-occupation fractions to fluctuate drastically during structural refinements. Consequently, very high quality diffraction data and/or complementary characterization techniques are needed in order to reliably determine the atomic structure of spinel iron oxide nanoparticles (Holder & Schaak, 2019 ▸). Indeed, our recent studies of the atomic structures of related nanocrystalline compounds in the spinel ferrite family have revealed substantial differences compared with the bulk equivalents (Andersen, Saura-Múzquiz *et al.*, 2018 ▸; Andersen *et al.*, 2019 ▸; Hölscher *et al.*, 2020 ▸). In addition, the surface of Fe_3_O_4_ particles is known to oxidize in air to form an Fe-deficient outer layer, commonly assumed to be γ-Fe_2_O_3_. Existing studies of solvothermally synthesized non-stoichiometric spinel iron oxide nanocrystallites evaluate the samples in terms of magnetite and maghemite content based on an idealized core-shell nanoparticle model (Salazar *et al.*, 2011 ▸; Frison *et al.*, 2013 ▸). However, we find no evidence of a Fe_3_O_4_/γ-Fe_2_O_3_ core-shell nanostructure being present based on the data in this study. Instead, we speculate that the nanoparticles are more likely to consist of a structurally coherent compositional gradient with an Fe-rich centre and an Fe-deficient outer region.

In the present study, we provide an in-depth characterization of the atomic and magnetic structure of spinel iron oxide nanoparticles with mean crystallite sizes in the range of ∼10–25 nm. The nanoparticles have been synthesized by sub-, near- and super-critical hydro­thermal treatment of an aqueous ammonium iron(III) citrate solution in a continuous-flow solvothermal reactor. The atomic and nanoscopic structure of the resulting nanoparticles is examined by joint Rietveld refinement of a constrained structural model to high-resolution synchrotron PXRD data and time-of-flight neutron powder-diffraction (NPD) data. In addition, the local atomic and magnetic ordering is examined by X-ray pair distribution function (PDF) analysis of X-ray and neutron total-scattering data. The neutron total-scattering data are modelled using a combination of conventional nuclear PDF analysis and magnetic PDF (mPDF) analysis, a recently developed method for the characterization of magnetic structures on the nanoscale (Frandsen *et al.*, 2014 ▸; Frandsen & Billinge, 2015 ▸). The mPDF method reveals both short-range and long-range magnetic correlations directly in real space and thus allows elucidation of the local magnetic ordering, as well as determination of the extent of the magnetic domains in the iron oxide nanoparticles. The scattering techniques are complemented by Mössbauer spectroscopy, transmission electron microscopy (TEM), scanning transmission electron microscopy (STEM), energy-dispersive X-ray spectroscopy (EDS) and the measurement of macroscopic magnetic properties by vibrating sample magnetometry yielding detailed insight into the size/structure/property relationship in the spinel iron oxide nanoparticle system.

## 2. Experimental   

### 2.1. Sample preparation   

The gram-scale preparation of surfactant/capping agent-free spinel iron oxide nanoparticles (generally necessary for characterization by NPD) was performed using the single-stage solvothermal flow synthesis apparatus at the Department of Chemistry, Aarhus University. The working principle of the apparatus is illustrated in the Supporting information and further details can be found elsewhere (Hald, 2009 ▸; Adschiri *et al.*, 1992 ▸; Hellstern *et al.*, 2015 ▸). The precursor solution was prepared by dissolving ammonium iron(III) citrate (C_6_H_8_O_7_ *x*Fe^3+^ *y*NH_3_, Sigma–Aldrich, reagent grade, 265.0 g mol^−1^) in deionized water to obtain a 0.1 *M* aqueous solution. Prior to the synthesis, the flow apparatus was pressurized with demineralized water to 250 bar, the solvent heater temperature was set to 370°C and the reactor was heated to the desired reaction temperature. In our previous studies, we have demonstrated how changing the reaction temperature in this system provides a handle for tuning the size and size distribution of the obtained iron oxide nanoparticles (Andersen *et al.*, 2014 ▸; Andersen, Bøjesen *et al.*, 2018 ▸; Jensen *et al.*, 2014 ▸). Consequently, three different hydro­thermal reaction conditions, *i.e.* 340°C (sub-critical, FL340C), 390°C (near critical, FL390C) and 440°C (super critical, FL440C), were used to obtain three samples with different particles sizes. The precursor and the solvent (demineralized water) were pumped separately into the reactor using air-driven liquid pumps. The precursor was pumped continuously at a rate of 5 ml min^−1^ and the preheated solvent was pumped continously at a rate of 15 ml min^−1^, giving a residence time of *ca.* 30 s in the reactor (*V*_reactor_ ≃ 10 ml). After exiting the reactor, the product was cooled and collected through a proportional release valve. The produced nanoparticles were separated from the solvent through centrifugation and subsequently washed repeatedly with demineralized water and ethanol. Finally, the surfactant-free nanopowders were dried in a vacuum oven at 60°C for 24 h. All material characterization was carried out within six months of the synthesis.

### 2.2. Powder X-ray diffraction and Rietveld refinement   

High-resolution synchrotron PXRD data were collected at the materials science beamline, BL44B2 (Adachi *et al.*, 2001 ▸), at SPring-8, Japan, using a large Debye–Scherrer camera. The X-ray wavelength was determined to be 0.50001 (1) Å (24.796 keV) by Rietveld refinement of a CeO_2_ standard (*a* = 5.411102 Å). The samples were loaded into 0.2 mm glass capillaries and the measurements were conducted at room temperature. Rietveld analysis was performed using the *FullProf* software package (Rodríguez-Carvajal, 1993 ▸). A joint refinement of a constrained structure model to the synchrotron PXRD patterns and time-of-flight NPD data (see below) was carried out. Details about the applied atomic structural model may be found in the Results and discussion[Sec sec3] and in the Supporting information. The PXRD and NPD backgrounds (including the diffuse scattering signal) were described by Chebychev polynomials, and the peak profiles were modelled using the Thompson–Cox–Hastings formulation of the pseudo-Voigt function (Thompson *et al.*, 1987 ▸). The instrumental contribution to the total peak broadening was determined by Rietveld refinement of data collected on a CeO_2_ calibrant powder in the same instrumental configuration and deconvoluted from the sample broadening in the refinements. The remaining sample contribution to the peak broadening was fitted assuming spherical strain-free crystallites. The mean volume-weighted diameter of the coherently scattering crystalline domains, 〈*D*〉, was estimated using the Scherrer formula, *H* = *K*λ/〈*D*〉 cos θ (Scherrer, 1918 ▸), where λ is the X-ray wavelength, θ is the Bragg scattering angle, *H* describes the peak broadening (full width at half the maximum intensity) and *K* is the shape factor, which was set to 0.829 assuming isotropic crystallite morphology (Langford & Wilson, 1978 ▸).

### 2.3. X-ray total scattering and PDF analysis   

Synchrotron X-ray total-scattering data were collected at the materials science beamline, ID11 (Kvick, 2003 ▸), at the European Synchrotron Radiation Facility (ESRF), Grenoble, France, for the FL340C and FL440C nanopowders packed in 1 mm borosilicate capillaries. An X-ray wavelength of 0.1897 Å (65.358 keV) was used giving a *Q*_max_ of 23.5 Å^−1^. The Fourier transformation of the X-ray total-scattering data into real-space PDFs was carried out using *PDFgetX3* (Juhás *et al.*, 2013 ▸), and the real-space structural refinements of the PDFs were conducted using *PDFgui* software (Farrow *et al.*, 2007 ▸). The experimental *Q*_damp_ (instrumental damping of PDF peak intensities) was determined to be 0.02895 Å^−1^ by refinement of National Institute of Standards and Technology (NIST) LaB_6_ 660B calibrant data collected in the same instrumental configuration. Additional details about the PDF data and model may be found in the Results and discussion[Sec sec3] and in the Supporting information.

### 2.4. Neutron total scattering and PDF/mPDF analysis   

Neutron total-scattering data were obtained at room temperature at the nanoscale-ordered materials diffractometer, NOMAD (Neuefeind *et al.*, 2012 ▸), at the Spallation Neutron Source (SNS), Oak Ridge National Laboratory, USA. The samples were loaded into 2 mm diameter quartz capillaries and data were measured at room temperature with an incoming wavelength spectrum of 0.1 to 3 Å. The raw total-scattering data in the *Q*_min_ to *Q*_max_ range from 0.5 to 25 Å^−1^ were reduced and Fourier transformed to produce the real-space PDF data using the beamline software. Because neutrons scatter from both the nuclei and the magnetic moments of the unpaired electrons, the experimental PDF patterns comprise both the atomic and mPDF components. The atomic PDF was modelled using the *PDFgui* software (Farrow *et al.*, 2007 ▸), while the mPDF modelling was performed with the *diffpy.mpdf* package, an extension of the *DiffPy-CMI* library (Juhás *et al.*, 2015 ▸).

### 2.5. Transmission electron microscopy   

TEM micrographs were measured on a Philips CM20 running at 200 kV with a LaB_6_ filament, while STEM micrographs were recorded on a FEI TALOS F200A analytical (S)TEM electron microscope equipped with an extreme field emission gun (X-FEG) electron source and a Ceta 16M camera. The STEM images were acquired using a high-angle annular dark-field detector and EDS elemental maps were obtained using a Super-X EDS detector. The samples were prepared by suspending a small amount of the dried product in 5 ml of ethanol and sonicating for 1 h. The sample was then evaporated onto a TEM grid at room temperature. The particle-size analysis was carried out using the program *Fiji* (Schindelin *et al.*, 2012 ▸), and quantitative analysis of the energy-dispersive X-ray spectra was carried out in the Bruker *ESPRIT* software suite.

### 2.6. Mössbauer spectroscopy   

Mössbauer experiments were performed using a standard transmission mode Mössbauer setup. An ∼5 mCi ^57^Co:Rh source was used. The system was calibrated relative to the centrum of α-Fe at room temperature.

### 2.7. Vibrating sample magnetometry   

The macroscopic magnetic properties of the samples were measured on cold-pressed pellets (diameter = 2.7 mm, mass ≃ 15 mg) using a quantum-design physical property measurement system equipped with a vibrating sample magnetometer (VSM). Zero-field-cooled/field-cooled (ZFC/FC) magnetization curves were measured in the temperature range from 10–300 K in an applied field of 40 kA m^−1^. In addition, field-dependent magnetization curves were recorded at 50 and 300 K, with the external magnetic field being scanned between ±2375 kA m^−1^. The VSM measurements were conducted with an oscillation frequency of 40 Hz and an averaging time of 2 s.

## 3. Results and discussion   

### 3.1. Crystal structure   

**Figure 3:**
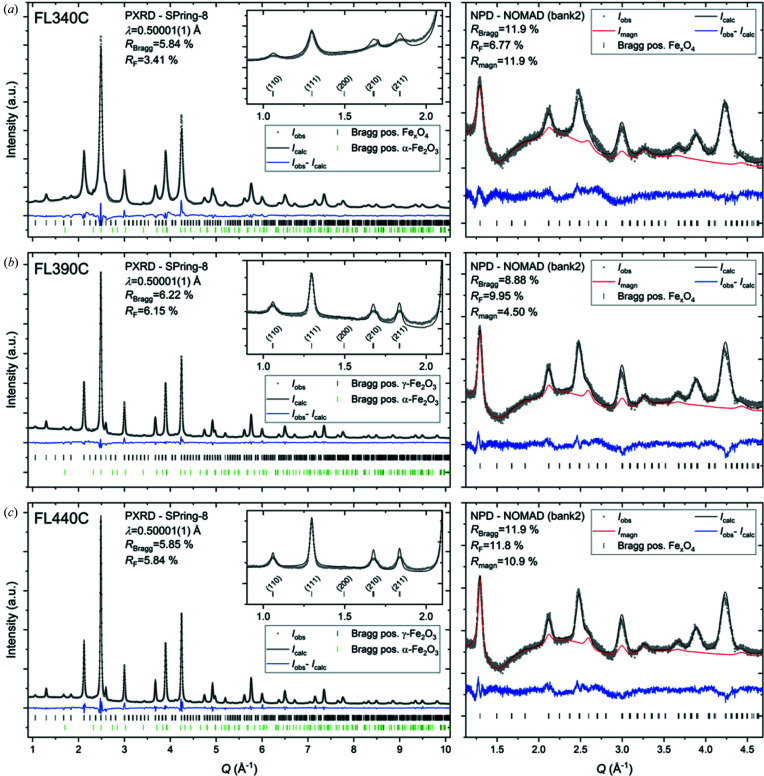
Joint refinements of SPring-8 high-resolution synchrotron PXRD data (left) and selected NOMAD time-of-flight NPD data (right) for the samples synthesized at (*a*) 340, (*b*) 390 and (*c*) 440°C. The additional NPD datasets from the other data banks and the corresponding fits may be found in the Supporting information. The insets show the low *Q* region of the PXRD data and illustrate the presence of the additional superstructure peaks arising from vacancy ordering in the structure. The main results obtained from the Rietveld analyses are summarized in Table 2 ▸ and further details about refined parameters may be found in the Supporting information.

Joint Rietveld refinements of a constrained structural model were carried out to the high-resolution synchrotron PXRD data and four time-of-flight NPD data banks for each sample. Fig. 3 ▸ shows the high-resolution synchrotron PXRD data and a selected time-of-flight NPD pattern (bank 2 is chosen to illustrate the main magnetic Bragg reflection at *Q* ≃ 1.3 Å^−1^) as well as the corresponding Rietveld fits for each of the nanocrystalline spinel iron oxide samples, FL340C, FL390C and FL440C, synthesized by the hydro­thermal flow method at 340, 390 and 440°C, respectively. The data from additional banks and corresponding fits can be found in the Supporting information. Notably, a minute amount (<1%) of hematite (α-Fe_2_O_3_, space group *R* 3 *c*) impurity was found in all three samples. For all samples, the dominating, most intense, peaks in the diffraction patterns can be indexed to the simple disordered spinel structure in 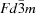. However, the high-resolution PXRD data clearly show the presence of superstructure peaks in the low *Q* region as illustrated in the insets of Fig. 3 ▸. For the FL340C sample, which was synthesized at the lowest temperature, the substantial peak broadening, as a result of the small crystallite size, makes it somewhat difficult to fully discern the superstructure peaks from the background. The positions of the superstructure peaks correspond to those seen for the structures in space group *P*4_3_32 or *P*4_3_2_1_2 (see Fig. 2 ▸), while the additional weak superstructure peaks characteristic of the *P*4_1_2_1_2 structure are not readily observed, thereby excluding this structural candidate. Considering the two remaining options, Rietveld refinements based on γ-Fe_2_O_3_ in space group *P*4_3_2_1_2 were found to yield the best match to the data, and the refined values of the tetragonal lattice parameters were close to the reported values of *a* = *b* ≃ 8.38 Å and *c* ≃ 8.34 Å. For the NPD data, the magnetic scattering contribution was refined in addition to the nuclear structure (see Fig. 3 ▸) using a collinear model with anti-parallel magnetic moment components along the crystallographic 〈111〉 direction refined as mean values on the tetrahedral and octahedral sites [μ_tet, oct_ (FL340) = 4.16 (9) μ_B_, μ_tet, oct_ (FL390) = 4.21 (5) μ_B_, μ_tet, oct_ (FL440) = 4.07 (10) μ_B_].

For all samples, the occupancy of each individual Fe site was initially refined. In these refinements, there was a clear tendency towards full occupancy of all Fe sites except for the octahedral Fe(4) 4*a* position [highlighted in red in Fig. 1 ▸(*c*)]. This is the same Fe vacancy site observed by Braun (1952 ▸), Greaves (1983 ▸) and Jørgensen *et al.* (2007 ▸), and confirms the vacancy ordering in the known maghemite structure. In the final refinements, the fractional occupancies of all other sites were thus kept fixed at full occupancy. Three different models for the Fe(4) 4*a* site occupancy were considered and compared to determine the robustness of the refinement of the occupancies. First, the site was kept fully occupied as would be seen in magnetite (Fe_3_O_4_). Second, the Fe(4) site occupation fraction was fixed at 0.333, as would be the case for a fully oxidized maghemite structure (γ-Fe_2_O_3_). Thirdly, the site occupancy was allowed to refine freely yielding a non-stoichiometric intermediate composition (Fe_*x*_O_4_). The obtained fits using the three different models for the three different samples are shown in the Supporting information. The refinement of the Fe(4) site occupation fraction consistently improved the agreement between data and model, and robustly refined to 0.583 (8), 0.604 (8) and 0.621 (7) for the FL340C, FL390C and FL440C samples, respectively. The refinements thus reveal a non-stoichiometric Fe_*x*_O_4_ composition of all samples which is between that of Fe_3_O_4_ and γ-Fe_2_O_3_. The non-stoichiometric composition and presence of mixed Fe^2+^/Fe^3+^ valence states in the samples were confirmed by analysis of Mössbauer spectra (see Table 2 ▸). The Mössbauer spectroscopy data and associated fits and analysis may be found in the Supporting information.

**Figure 4:**
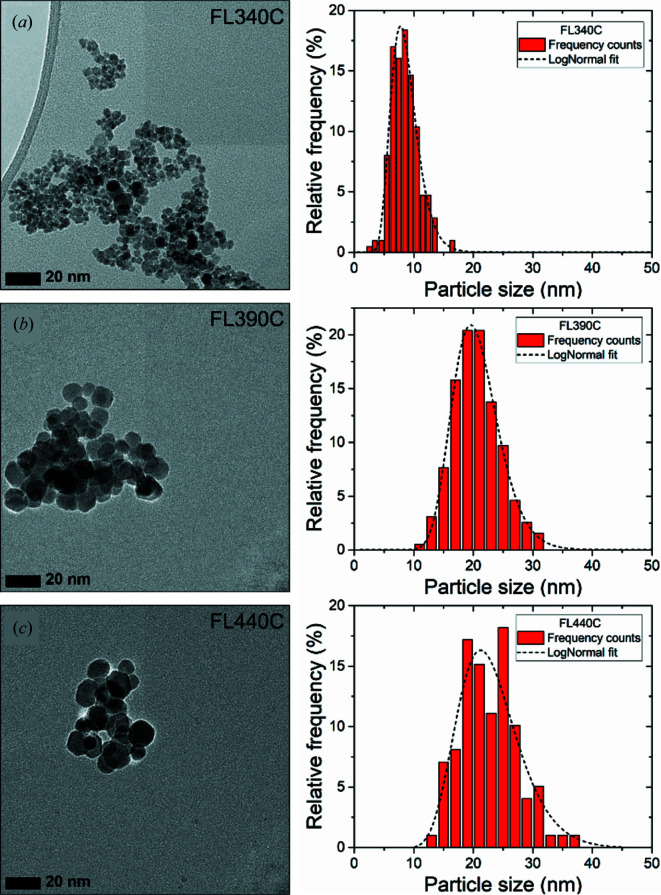
Representative TEM images and corresponding size analyses of the three iron oxide nanoparticle samples prepared at (*a*) 340, (*b*) 390 and (*c*) 440°C. The histograms have been fitted by a lognormal size distribution, as indicated by the dashed black line. Additional TEM images may be found in the Supporting information.

The Rietveld refinement with Scherrer size analysis of the PXRD data yielded crystallite sizes of 8.81 (9), 17.4 (3) and 25.4 (3) nm for the FL340C, FL390C and FL440C samples, respectively. The microstrain contribution to the peak broadening was found to be negligible as the associated parameters tended to zero when included in the refined model (see the Supporting information). As expected, based on our previous *in situ* PXRD and PDF studies of the hydro­thermal formation of iron oxide nanoparticles (Andersen *et al.*, 2014 ▸; Andersen, Bøjesen, *et al.*, 2018 ▸; Jensen *et al.*, 2014 ▸), a higher reaction temperature leads to an increase in the final crystallite sizes. Notably, when inspecting the data and fits in detail it is seen that the profiles of the superstructure peaks for all three samples are significantly broader than the main Bragg peaks (see the insets of Fig. 3 ▸). In fact, for the smallest nanoparticles, FL340C, only very weak broad features are seen where superstructure peaks are expected. Consequently, it is difficult for the model to accurately match the superstructure line profile. If applying Scherrer analysis on the (210) and (211) superstructure peaks alone, superstructure domain sizes of 8 (1), 10 (2) and 10 (3) nm are extracted from the FL340C, FL390C and FL440C samples, respectively (see the Supporting information for details). This result shows that the superstructure domains with Fe site vacancy ordering are smaller than the full crystallite size and/or contain anti-phase boundaries, faulting or other disorder effects. Studying the structure of non-stoichiometric spinel iron oxide nanoparticles synthesized from Fe^III^ and Fe^II^ containing precursors, Frison *et al.* hypothesized a core/shell nanoparticle structure, where an Fe_3_O_4_ core is surrounded by an oxidized γ-Fe_2_O_3_ layer owing to air exposure (Frison *et al.*, 2013 ▸). Such a system would result in a reduced intensity of the superstructure peaks as only part of the particle consists of maghemite. A similar model could be expected for our current system; however, attempts at implementing a two-phase model based on Fe_3_O_4_ in space group 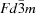 and γ-Fe_2_O_3_ in space group *P*4_3_2_1_2 [fixing the Fe(4) 4*a* site occupation fraction to 0.33] did not yield a satisfactory fit. As illustrated in the insets in Fig. 3 ▸, the observed intensities of the (210) and (211) superstructure peaks in the PXRD data are very similar, but the (210) reflection would clearly be the more intense of the two in vacancy-ordered γ-Fe_2_O_3_ (space group *P*4_3_2_1_2), as illustrated in the simulated PXRD pattern in Fig. 2 ▸(*c*). Therefore, a two-phase model (be it core-shell type or distinct particles) based on a combination of stoichiometric Fe_3_O_4_ and γ-Fe_2_O_3_ phases will not be able to fit the data. Given the near-isotropic morphology of the nanoparticles (see the TEM images in Fig. 4), this discrepancy in relative peak intensities is unlikely to originate from either texture or anisotropic crystallite morphology. Consequently, the vacancy-ordered Fe_*x*_O_4_ model with intermediate non-stoichiometric composition (as described above) needs to be employed, in order to account for this discrepancy in relative intensities. The lack of a Fe_3_O_4_ core is further supported by the absence of the characteristic Verwey transition in thermomagnetic data [see Section 3.4 and Fig. 6(*a*)], as well as the lack of a discontinuous Fe_3_O_4_/γ-Fe_2_O_3_ core-shell transition in STEM-EDS line scans collected on individual particles (see the Supporting information). Instead of the ideal core-shell model, we speculate that the powder-diffraction patterns are best rationalized by a nanoparticle structure consisting of a structurally coherent gradual transition from a relatively Fe-rich core to a more oxidized outer region, with a net Fe/O stoichiometric ratio in between that of Fe_3_O_4_ and γ-Fe_2_O_3_.

### 3.2. Nanostructure   

Representative TEM micrographs for the samples are shown in Fig. 4 ▸. The TEM images confirm that the prepared iron oxide nanoparticles have close to isotropic morphology. Some particles, especially the larger ones, exhibit a hexagonally shaped projection [see, for example, Fig. S15(*c*) in the Supporting information], which could indicate a cubic, octahedral or even icosahedral morphology. For each of the samples, size analysis was carried out by measuring the dimensions of a representative number of particles in TEM micrographs collected at different places on the grids. The resulting particle-size histograms are shown in Fig. 4 ▸ next to the corresponding TEM images. The histograms were fitted by a lognormal size distribution from which the mean particle size and standard deviation were determined, and the number-averaged particle sizes from the TEM analysis (〈*D*_TEM_〉) are listed in Table 2 ▸. For comparison, the mean volume-weighted crystallite sizes from the Scherrer analysis of the PXRD data (〈*D*_PXRD_〉) are also listed in the table ▸. A relatively good agreement is observed between the PXRD and TEM sizes. Notably, the magnetic nature of the particles along with the absence of surfactants cause them to aggregate making it somewhat difficult to distinguish the individual particles. This is particularly true for smaller particles in the aggregate, which may explain the larger 〈*D*_TEM_〉 values. Another common issue in comparing crystallite sizes from PXRD analysis with particle sizes from TEM is that the PXRD analysis gives the mean size of coherently scattering crystalline domains while TEM analysis typically gives the size of entire particles, which may consist of several crystalline domains. In addition, amorphous regions are not accounted for in the PXRD analysis, which again may lead to smaller sizes compared with TEM (Weidenthaler, 2011 ▸). In any case, the overall trend of increasing particle size with synthesis temperature is consistent with the PXRD analysis.

### 3.3. X-ray PDF analysis   

**Figure 5:**
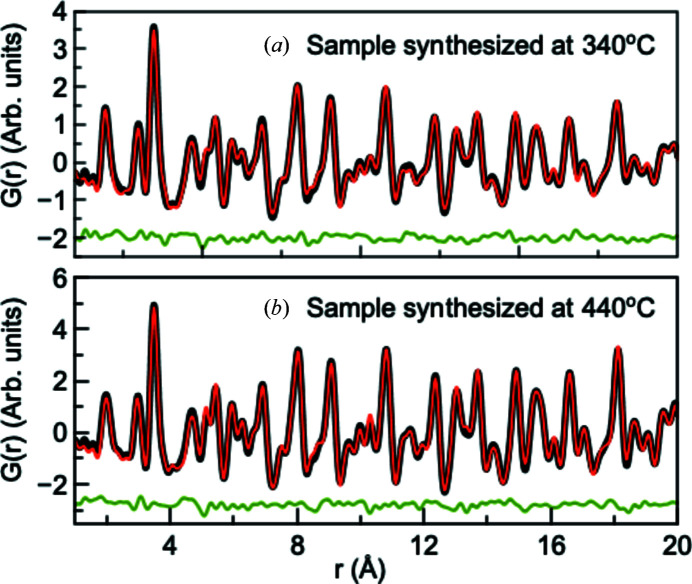
Experimental PDFs (black), PDFs calculated from fitted models (red) and difference curves (blue) for the spinel iron oxide nanoparticles synthesized at (*a*) 340 and (*b*) 440°C. Fits including the 1–60 Å range can be found in Fig. S17.

In the context of nanosized samples, X-ray total-scattering experiments may yield further structural information than standard Rietveld refinements in *Q* space (Billinge & Kanatzidis, 2004 ▸). Fig. 5 ▸ shows PDFs obtained from X-ray total-scattering data measured at ID11, ESRF, for the two samples synthesized at *T* = 340°C and *T* = 440°C along with the refined model. Fits of the *G*(*r*) function using a wider *r* range (1–60 Å) are shown in Fig. S17.

The fits over the 1–60 Å range were initially carried out with one single crystalline γ-Fe_2_O_3_ model. However, as shown in Fig. S17, this model did not fully describe the peak intensities in the high *r* region, as is especially clear for sample FL340 containing the smallest particles. This type of misfit can be seen for samples with high size polydispersity, and a model with a lognormal crystallite size distribution was initially tested which, however, did not converge to physical values for size and size distribution and did not improve the fit quality. Instead, when fitting the 1–60 Å range of the PDF from sample FL340, the PDF was modelled with two maghemite phases with different crystallite sizes, which significantly improved the fit, lowering the *w* *R* values from 16 to 11%, indicating a very broad distribution of crystallite sizes. The unit-cell parameter and atomic displacement parameters were refined independently for the two phases, while the fractional atomic coordinates and occupancy of the Fe(4) site were constrained to take the same values for both phases. The two-phase fit to the PDFs from the FL340 sample indicates a high percentage (*ca*. 50%) of a nanostructured phase, where the crystallite size refines to *ca.* 2.5 nm. The presence of such small crystallites may be expected from the TEM results in Fig. 4 ▸.

The occupancies of all four iron atoms were initially allowed to vary, but only that of Fe(4) was refined to partial occupancy, in accordance with the *Q*-space Rietveld refinement. However, no matter the *r* range included in the refinement, the PDF refinements indicate that the synthesis temperature to a larger extent affects the vacancy concentration and thus the Fe_*x*_O_4_ stoichiometry. The Fe(4) site occupancy obtained from the real-space PDF analysis of the sample synthesized at 440°C is slightly higher than that obtained from the *Q*-space refinements, while the refined occupancy on the Fe(4) site in the 340°C sample is lower in the PDF fitting giving a net stoichiometry of *x*_PDF_ ≃ 2.70 and *x*_PDF_ ≃ 2.85, respectively. A complete compilation of the refined parameters may be found in the Supporting information. When inspecting the fit quality in Figs. 5 ▸ and S17, a small but structured residual is seen to remain over the entire PDF *r* range for both samples. The fit quality did not improve on incorporation of hematite or other iron oxide or hydroxide phases, and the misfit may arise from the non-stochiometric nature of the samples where the model in space group *P*4_3_2_1_2 does not fully capture the structural details.

### 3.4. Magnetic properties   

At ambient conditions, Fe_3_O_4_ and γ-Fe_2_O_3_ both exhibit soft magnetic properties arising from a ferrimagnetic ordering of their atomic moments induced by an anti-ferromagnetic superexchange coupling between the magnetic Fe ions in the tetrahedral and octahedral spinel sublattices. A net magnetic moment is obtained by the incomplete cancellation of the magnetism of the two lattices owing to the surplus of octahedrally coordinated species (Chikazumi & Graham, 2009 ▸), and, given its larger octahedral occupancy, the Fe_3_O_4_ phase thereby attains a higher saturation magnetization (∼92 A m^2^ kg^−1^) than γ-Fe_2_O_3_ (∼84 A m^2^ kg^−1^) (Coey, 2010 ▸). Reducing their particle size below the superparamagnetic limit leads to zero net magnetization in zero-field conditions, at ambient temperature, owing to thermally induced random spin reorientations (Kodama, 1999 ▸; Lu *et al.*, 2007 ▸). Notably, the magnetic ordering in stoichiometric Fe_3_O_4_ famously changes from the cubic spinel structure to a monoclinic structure when cooled below *T*_V_ ≃ 125 K (Wright *et al.*, 2002 ▸; Senn *et al.*, 2012 ▸). This is called the Verwey transition and occurs because of a cease of the minority spin ‘extra’ electrons hopping between the octahedrally coordinated Fe^3+^ and Fe^2+^ ions and the concomitant charge ordering of the two ionic species in the structure (Walz, 2002 ▸; Senn *et al.*, 2012 ▸). In a recent study, PDF analysis of multitemperature X-ray total-scattering data was used to demonstrate how local fluctuations in the Fe–Fe bonding in stoichiometric Fe_3_O_4_ is the primary cause of the electronic instability giving rise to the Verwey transition (Perversi *et al.*, 2019 ▸). The Verwey transition is readily observed in thermomagnetic curves as a spontaneous jump in the magnetization, even for nanocrystalline samples (Özdemir *et al.*, 1993 ▸). The highest Verwey-transition temperature is observed for pure Fe_3_O_4_ and it is effectively suppressed by even small deviations from this stoichiometry (Aragón *et al.*, 1985 ▸). Consequently, the presence of a Verwey transition would be indicative of stoichiometric Fe_3_O_4_ domains being present in the sample.

**Figure 6:**
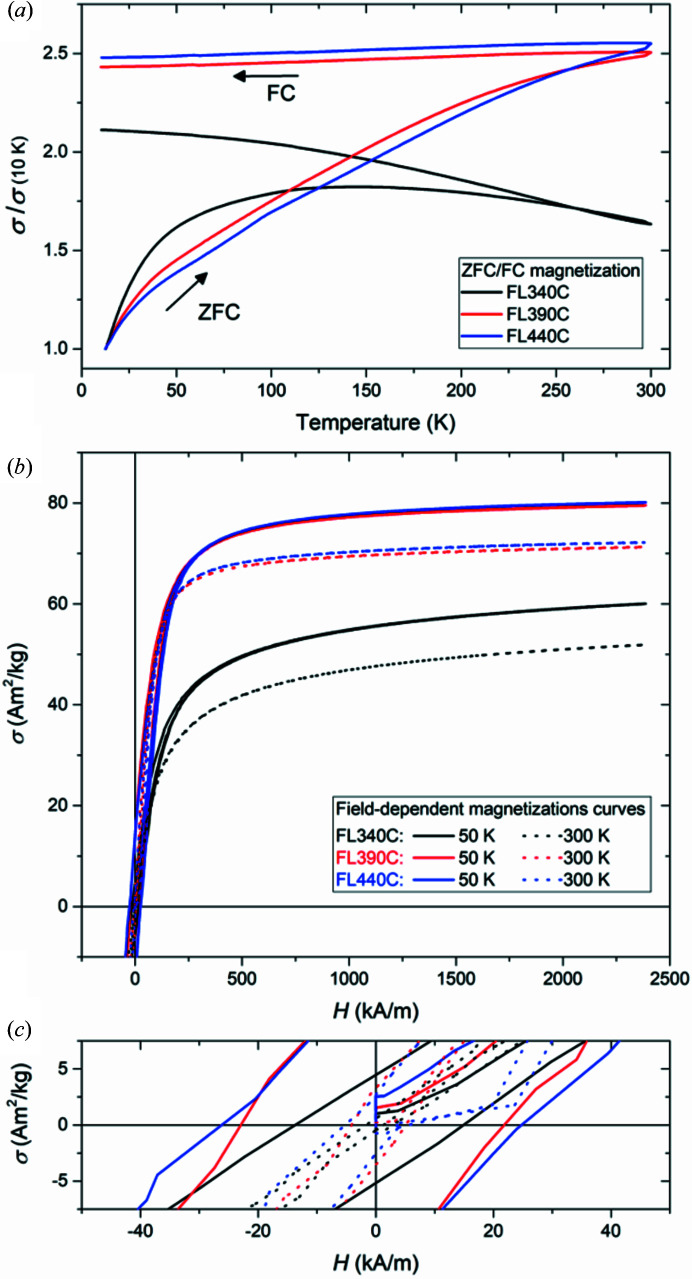
(*a*) Normalized ZFC/FC magnetization curves in the 10–300 K temperature range in an applied field of 40 kA m^−1^. (*b*) Field-dependent magnetization curves of the nanopowders collected using a VSM at 50 K (solid lines) and 300 K (dashed lines). (*c*) An enlarged view of the low-field region illustrating the difference in the coercive fields of the samples.

Fig. 6 ▸(*a*) shows the ZFC/FC magnetization curves for the three samples. For all three samples, the absence of stoichiometric Fe_3_O_4_ domains is corroborated by the lack of the characteristic Verwey-transition feature in the thermomagnetic data. Notably, *T*_V_ has been reported to gradually decrease with decreasing particle size and to disappear for very small particles (<10 nm) (Goya *et al.*, 2003 ▸). However, more recent studies of the thermomagnetic behaviour report observation of the Verwey transition for non-stoichiometric spinel iron oxide particles containing Fe_3_O_4_ domains below <10 nm (*T*_V_ ≃ 95 K) (Salazar *et al.*, 2011 ▸) and for Fe_3_O_4_ crystallites as small as 6 nm (*T*_V_ ≃ 120 K) (Mitra *et al.*, 2014 ▸). The absence of the Verwey transition in the FL340C sample, which comprises the smallest crystallites (<10 nm), could thus potentially be ascribed to its reduced size. The transition from the blocked to superparamagnetic state of the FL340C sample is observed in the ZFC curve as a broad peak. The larger crystallites of the FL390C and FL440C samples seem to approach their blocking temperature, but full unblocking of the particles has yet to be attained at room temperature.

In addition, field-dependent mass magnetization measurements were carried out at both 300 and 50 K, see Fig. 6 ▸(*b*). The FL390C and FL440C samples exhibit very similar magnetic behaviours at both temperatures, while FL340C displays substantially lower magnetization. This is probably a result of the lower Fe^2+^ content in the positively contributing octahedral sublattice of the FL340C nanocrystallites, as well as being an effect of the smaller mean crystallite size. Small nanoparticles often exhibit a reduced saturation magnetization owing to surface spin disorder, reduced crystallinity or structural defects (Nedelkoski *et al.*, 2017 ▸; Mørup *et al.*, 2013 ▸). The macroscopic mass-specific saturation magnetizations, σ_s, VSM_, were obtained from the curves by extrapolation using the law of approach to saturation (Brown, 1941 ▸) and are summarized in Table 3 ▸. The observed room-temperature mass saturation magnetizations are all below the intrinsic bulk values of both Fe_3_O_4_ (∼92 A m^2^ kg^−1^) and γ-Fe_2_O_3_ (∼84 A m^2^ kg^−1^) as a result of the small particle size.

The curves all exhibit the characteristic S shape of a soft magnetic material. At 300 K, all three samples exhibit a negligible coercive field, as illustrated in Fig. 6 ▸(*c*), indicating superparamagnetic behaviour. However, reducing the temperature to 50 K causes a slight opening of the hysteresis loop, which seems to correlate with crystallite size. For fine superparamagnetic particles, the time between random spin reversals, τ, is given by the Néel–Brown law, τ = τ_0_ exp(*K*_1_ *V*/*k*_B_ *T*), where τ_0_ is the attempt time (∼1 ns), *K*_1_ is the magnetocrystalline anisotropy constant, *k*_B_ is the Boltzmann constant, *T* is the temperature (Skomski, 2003 ▸) and *V* is the crystallite volume. By setting the flipping time equal to the averaging time of the measurement, τ_M_, the critical superparamagnetic threshold dimension, *D*_SP_, can be estimated by *D*_SP_ = [−6*k*_B_ *T* ln(τ_M_/τ_0_)/(π*K*_1_)]^1/3^. Based on intrinsic parameters of Fe_3_O_4_ (*K*_1_ = −0.011 MJ m^−3^) (Skomski, 2003 ▸), a *D*_SP_ at 300 K of 24.3 nm and at 50 K of 13.4 nm can be estimated. Equivalently for γ-Fe_2_O_3_, a *D*_SP_ at 300 K of 32.5 nm and at 50 K of 17.9 nm is calculated. Consequently, the magnetic behaviours of the samples are in good agreement with the size analyses discussed earlier.

### 3.5. Local magnetic structure – mPDF   

Experimental PDFs were also obtained from neutron total-scattering data collected at room temperature on the NOMAD instrument at the SNS. Fits were initially performed over the data range 1.5–50 Å using a single phase in the *P*4_3_2_1_2 γ-Fe_2_O_3_ structure. The Fe(4) occupancies for FL340C, FL390C and FL440C are 0.5 (1), 0.8 (1) and 0.9 (1), respectively, corroborating the previous observation that FL340C is closer to γ-Fe_2_O_3_ composition (containing only Fe^3+^ ions) while FL390C and FL440C have a significant Fe^2+^ component. The refined spherical particle diameters for FL340C, FL390C and FL440C determined by the neutron PDF fits are 11 (1) nm, 24 (1) nm and 28 (1) nm, respectively, confirming the size trend revealed by TEM, PXRD and X-ray PDF.

**Figure 7:**
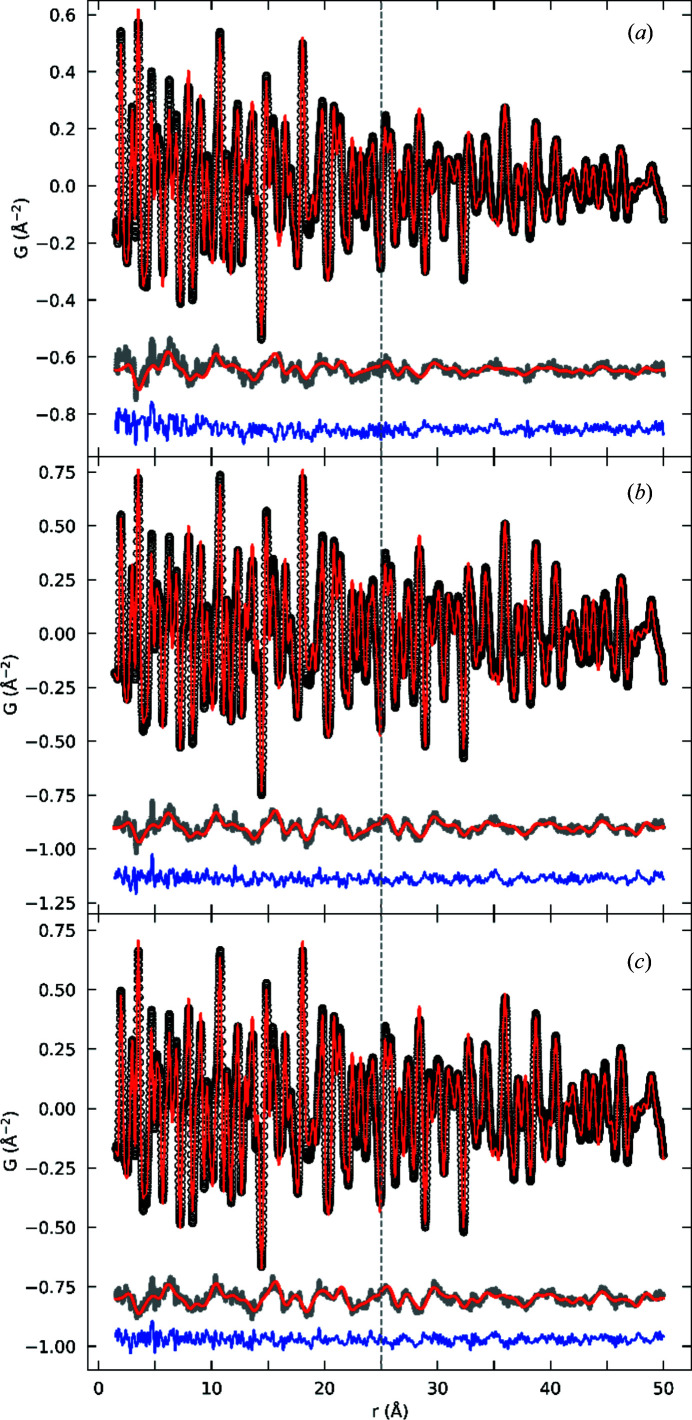
Neutron PDFs obtained from NOMAD data for the three samples synthesized at (*a*) 340, (*b*) 390 and (*c*) 440°C. The data (black) are fitted with the *P*4_3_2_1_2 model (red). The difference curve (grey) shows clear oscillations for all three samples owing to well defined magnetic correlations. The red curves overlaid on the grey curves represent the mPDF fits described in the text. The total fit residual after including both the atomic and mPDF components is given by the blue curve in each panel. The vertical dashed line at *r* = 25 Å marks the division between the two fitting ranges used (1.5–25 and 25–50 Å).

As has been shown elsewhere (Olds *et al.*, 2018 ▸), PDF data collected on NOMAD result in an artificial *r-*dependent variation in the lattice parameters, as well as both *Q*- and *r*-dependent instrumental resolution functions. This causes fits performed over data ranges larger than ∼30 Å to suffer in quality. Accordingly, we performed a second set of fits in which the data were broken into two ranges, 1.5–25 Å and 25–50 Å, allowing more quantitatively accurate fits. To reduce correlations and for simplicity, only one phase was included in the fits, and occupancies of Fe(4) were fixed to the values determined from the X-ray PDF fits. Fig. 7 ▸ shows the results of these fits, with the dashed vertical line marking the division between the two fitting ranges. The fits match the experimental PDF well, but close inspection of the difference curve (the grey curve immediately below the fit) reveals an oscillatory signal rather than just random noise. Neutrons probe not only atomic structure but also magnetic structure, and the observation of oscillations in the difference curve is a clear sign of the presence of a magnetic structure with well defined magnetic correlations, as also evident from the reciprocal-space data from bank 2 in Fig. 3 ▸. A magnetic component was therefore added to the total PDF model to describe the magnetic contribution to the signal. Assuming that the magnetic structure within each nanoparticle is similar to the ferrimagnetic structure of bulk Fe_3_O_4_ (Coey, 2010 ▸), we constructed a model consisting of one magnetic sublattice for the octahedral Fe sites and another sublattice for the tetrahedral Fe sites and we independently refined the spin magnitude and direction of each sublattice. The size of the magnetic domains was further refined by applying a spherical-shape function similar to that used for the atomic PDF. As has been carried out previously for materials carrying a net magnetic moment, we included a term linear in *r* with a negative slope whose magnitude is proportional to the net magnetic moment in the calculations of the mPDF (Frandsen *et al.*, 2016 ▸; Kodama *et al.*, 2017 ▸). The atomic and mPDF refinements were performed sequentially, *i.e.* we first refined the atomic structure and then used the resulting difference curve as the ‘data’ for the magnetic refinement.

The resulting magnetic fits are displayed as the red curves overlaid on the grey difference curves in Fig. 7 ▸. The model describes the features in the difference curve well, leaving a relatively flat total difference curve for each sample (the blue curves in Fig. 7 ▸). For each sample, the spin directions of the two sublattices robustly refined to be antiparallel to each other, in agreement with the bulk magnetic structure. At room temperature, the magnetic easy axes, *i.e.* collinear atomic magnetic dipole moments, order in bulk γ-Fe_2_O_3_ and Fe_3_O_4_ along the crystallographic 〈111〉 direction (Coey, 2010 ▸). However, we note that the present analysis did not allow reliable discrimination between ordering along the 〈111〉, 〈100〉 or 〈110〉 directions to be obtained and thus the spins were fixed to be in the 〈111〉 bulk-like direction in the final refinement. In each case, the refined magnetic domain size was somewhat smaller than the particle diameter, as determined by the atomic PDF fits (see Table 3 ▸). Deviations from the magnetic order near the surface of the particle could contribute to the reduced magnetic domain size as the magnetic moments are spin canting at the surface (Mørup *et al.*, 2013 ▸; Nedelkoski *et al.*, 2017 ▸; Kodama *et al.*, 1996 ▸). The refined magnetic moments are also provided in Table 3 ▸ and are generally consistent with values extracted from the reciprocal-space refinements of the neutron data and values reported in the literature (Wright *et al.*, 2000 ▸). We note that the magnetic domain size was determined from fits over the data range 1.5–50 Å, which was more suitable for probing the real-space damping of the mPDF signal than a smaller fitting range would have been.

In summary, the neutron PDF data confirm the trends in particle size and Fe(4) occupancy revealed by the X-ray PDF results, while also enabling real-space modelling of the magnetic structure via mPDF analysis. The ferrimagnetic structure observed in bulk maghemite and magnetite provides a quantitatively accurate fit to the mPDF data, and the refined magnitudes of the magnetic moments on the tetrahedral and octahedral sites are comparable with the bulk values. Interestingly, the best-fit magnetic domain sizes for the three different samples are ∼60–70% of the best-fit nanoparticle size, indicating that perfect magnetic coherence does not extend through the entire nanoparticle. More generally, the success of the mPDF analysis shown here motivates further studies of magnetic nanoparticle systems using this technique.

## 4. Conclusions   

The atomic structure of spinel iron oxide nanoparticles synthesized by the flow hydro­thermal method has been meticulously analysed by structural modelling of synchrotron X-ray and neutron powder-diffraction and total-scattering data. It is observed that a non-stoichiometric structural model based on a tetragonal γ-Fe_2_O_3_ phase with vacancy ordering in the structure (space group *P*4_3_2_1_2) yields the best fit to the powder X-ray diffraction and total-scattering data. Notably, this tetragonal structure features vacancies on only one of three distinct octahedral metal sites in the structure. Interestingly, it is observed that the coherency length of the vacancy-ordered domains in the structure is smaller than the total size of the crystallites. Previous studies dealing with non-stoichiometric spinel iron oxide nanoparticles have suggested a core-shell nanoparticle structure consisting of an Fe_3_O_4_ core and an oxidized γ-Fe_2_O_3_ shell. However, in the present study, we found no evidence of any distinct stoichiometric Fe_3_O_4_ or γ-Fe_2_O_3_ domains present in the samples. Thermomagnetic data show no signs of the characteristic Verwey transition of stoichiometric Fe_3_O_4_, and STEM-EDS line scans across individual particles show no discontinuous steps in elemental composition. Furthermore, a two-phase model containing stoichiometric components of Fe_3_O_4_ and vacancy-ordered γ-Fe_2_O_3_ does not satisfactorily fit the X-ray and neutron scattering data. Instead, we speculate that the examined particles are more likely to consist of a structurally coherent compositional gradient going from a Fe-rich vacancy-ordered centre towards a more oxidized and disordered outer region. We note that different synthesis methods may result in different atomic structures with different mean iron-oxidation states. Consequently, meticulous structural characterization in each individual case is crucial for obtaining a deeper understanding of the structure–property relationship and thereby rationally designing improved magnetic nanomaterials. Finally, the local magnetic structure of the nanoparticles was determined using the recently developed mPDF method revealing a ferrimagnetic ordering with magnetic domain sizes of ∼60–70% of the total nanoparticle size. This is the first study in which mPDF analysis has been applied to magnetic nanoparticles, establishing a successful precedent for future studies of nanosized magnetic systems using this technique.

## Supplementary Material

Supporting information. DOI: 10.1107/S2052252520013585/lt5032sup1.pdf

## Figures and Tables

**Table 1:** The atomic structural model in tetragonal space group *P*4_3_2_1_2 [as reported by Greaves (1983 ▸)] employed in the refinement of the FL440C sample The corresponding refined parameters for the FL340C and FL390C samples may be found in the Supporting information. The numbers in parentheses indicate the errors on the last significant digit of the refined parameters. The secondary hematite phase (space group *R* 3 *c*) was found to constitute 0.7 (5) wt%. *B*_iso, overall_ is a common isotropic atomic displacement parameter refined for all atoms.

FL440C (Fe_*x*_O_4_)					
Space group: *P*4_3_2_1_2 (No. 96), *a* = *b* = 8.3846 (4) Å, *c* = 8.3461 (4) Å, α = β = γ = 90°, *B*_iso, overall_ = 0.26 (1) Å^2^					
Atom	Site	*x*	*y*	*z*	Site occupation fraction
Fe1	8*b*	0.746 (2)	0.996 (2)	0.121 (1)	1
Fe2	4*a*	0.621 (1)	*x*	0	1
Fe3	8*b*	0.367 (1)	0.869 (1)	−0.010 (1)	1
Fe4	4*a*	0.131 (2)	*x*	0	0.621 (7)
O1	8*b*	0.615 (4)	0.865 (5)	−0.012 (4)	1
O2	8*b*	0.115 (5)	0.380 (6)	−0.007 (4)	1
O3	8*b*	0.132 (6)	0.876 (6)	0.008 (4)	1
O4	8*b*	0.383 (5)	0.627 (5)	0.003 (3)	1

**Table 2:** The main results from combined Rietveld refinements of PXRD and NPD data along with size analyses and obtained stoichiometries from various techniques The Rietveld refinements and PDF analyses were carried out based on the atomic structure in space group *P*4_3_2_1_2 with full occupancy on all other sites than Fe 4*a*. sp-*D* = refined spherical particle diameter, XPDF = X-ray PDF, NPDF = neutron PDF.

	FL340C	FL390C	FL440C
Structure (PXRD + NPD)			
*a* (Å)	8.3838 (8)	8.3784 (2)	8.3846 (4)
*c* (Å)	8.3452 (10)	8.3458 (4)	8.3461 (4)
Fe(4) site occupancy fraction	0.583 (8)	0.604 (8)	0.621 (7)
Crystallite/particle size			
〈*D*_PXRD_〉 (nm)	8.81 (9)	17.4 (3)	25.4 (3)
〈*D*_superstructure_〉 (nm)	8 (1)	10 (2)	10 (3)
〈*D*_(220)_〉 (nm)	9.4 (3)	19 (1)	20.5 (6)
〈*D*_TEM_〉 (nm) ± standard deviation (nm)	8.63 (4) ± 2.3 (7)	20.7 (4) ± 4.0 (7)	23.0 (3) ± 5.4 (7)
〈sp-*D*_XPDF, phase1_〉 (nm)	11.0	N/A	13.4†
〈sp-*D*_XPDF, phase2_〉 (nm)	2.6	N/A	–
〈sp-*D*_NPDF, nuclear_〉 (nm)	11.0 (2)	24 (1)	28 (1)
Stoichiometry			
Fe_*x*_O_4_, *x* (PXRD + NPD)	2.791 (4)	2.802 (4)	2.811 (3)
Fe_*x*_O_4_, *x* (X-ray PDF)	2.70	N/A	2.85
Fe_*x*_O_4_, *x* (Mössbauer)	2.69 (6)	2.71 (2)	2.71 (3)

† PDF sizes of large crystallites are probably underestimated owing to the rapid acquisition PDF (RA-PDF) geometry applied for X-ray total-scattering measurements.

**Table 3:** Macroscopic magnetic properties measured by a VSM, and refined mPDF parameters for the magnetic structure

	FL340C	FL390C	FL440C
VSM			
σ_s, VSM_ 50 K (A m^2^ kg^−1^)	62.6 (2)	80.5 (1)	81.2 (3)
σ_s, VSM_ 300 K (A m^2^ kg^−1^)	54.4 (2)	72.2 (1)	73.1 (3)
Rietveld (PXRD + NPD)			
μ_tet, oct_ (average) (μ_B_)	4.16 (9)	4.21 (5)	4.07 (10)
mPDF			
μ_tet_ (μ_B_)	3.9 (1)	4.2 (1)	4.2 (1)
μ_oct_ (μ_B_)	4.3 (1)	3.6 (1)	3.6 (1)
Nuclear spherical domaindiameter (nm)	11.0 (2)	24 (1)	28 (1)
Magnetic spherical domaindiameter (nm)	8 (1)	15 (2)	18 (6)
