# Supplementary material for: Local and long-range atomic/magnetic structure of non-stoichiometric spinel iron oxide nanocrystallites
Source: IUCrJ. 2021 Jan 1;8(Pt 1):33–45. doi: 10.1107/S2052252520013585 (PMC7792993; doi:10.1107/S2052252520013585)
Supplement: Supplementary file 1 [file m-08-00033-sup1.pdf]

IUCrJ

Volume 8 (2021)

Supporting information for article:

Local and long-range atomic/magnetic structure of non-stoichiometric spinel iron oxide nanocrystallites

Henrik L. Andersen, Benjamin A. Frandsen, Haraldur P. Gunnlaugsson, Mads R. V. Jørgensen, Simon J. L. Billinge, Kirsten M. Ø. Jensen and Mogens Christensen

S1. Continuous solvothermal flow synthesis setup

The design of the employed continuous flow reactor at Department of Chemistry, Aarhus University, is illustrated in Fig. S1. The prepared precursor is initially loaded into the 180 ml injector column which is connected to the reactor through Swagelok™ tubing and fittings. An injection pump pumps water into the injector column and thereby pushes down a piston, which in turn forces the precursor out the other end of the injector and into the pressurized reactor (≤250 bar). The precursor is pumped at a rate of around 5 ml/min and it thus generally takes 30-40 minutes for the reaction of a full injector (180 ml). The precursor must be dilute (<0.1 M) since crystallization from a highly concentrated precursor tends to clog up the tubing. The solvent is pumped into the system through a second line, and can be preheated up to 450 °C. The precursor is mixed with the preheated solvent at the T-piece, which leads to a very rapid heating, and an immediate nucleation of a large number of primary nuclei is induced. Subsequently, the mixture flows through the heated reactor column (Inconel625, 50 cm long, 4.3 mm inner diameter) where further crystallization and growth of the particles take place. Ultimately, the reaction is quenched and the product collected through a proportional release valve (PRV). The flow setup provides several handles for tuning the physical reaction parameters and consequently vary the obtained product characteristics. These include the relative solvent and precursor flow rates (reaction time, heating rate), preheated solvent temperature (heating rate), reactor temperature and reactor pressure.

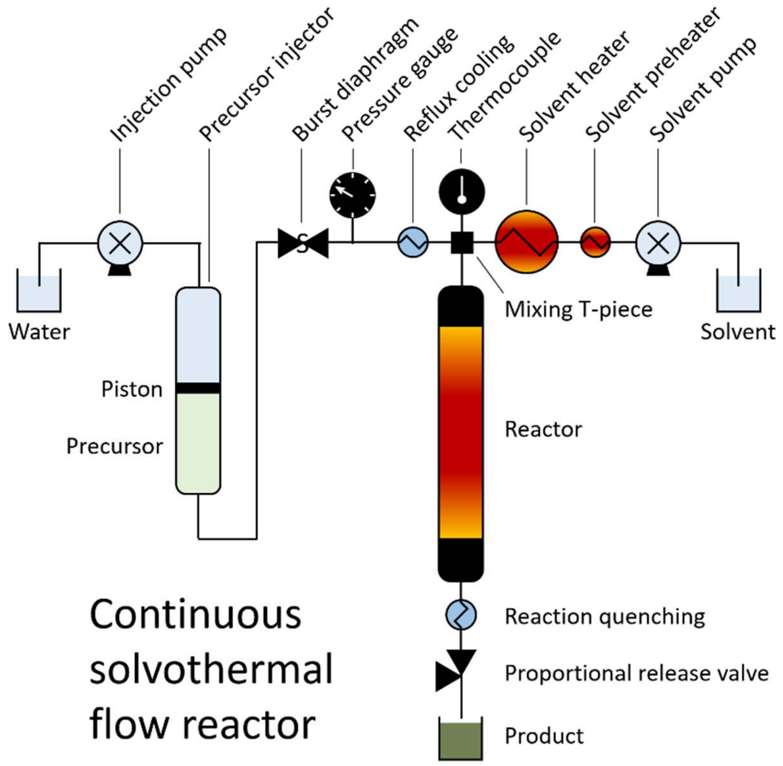

Figure S1 Diagram of the employed continuous solvothermal flow reactor.

S2. PXRD – FL340C models

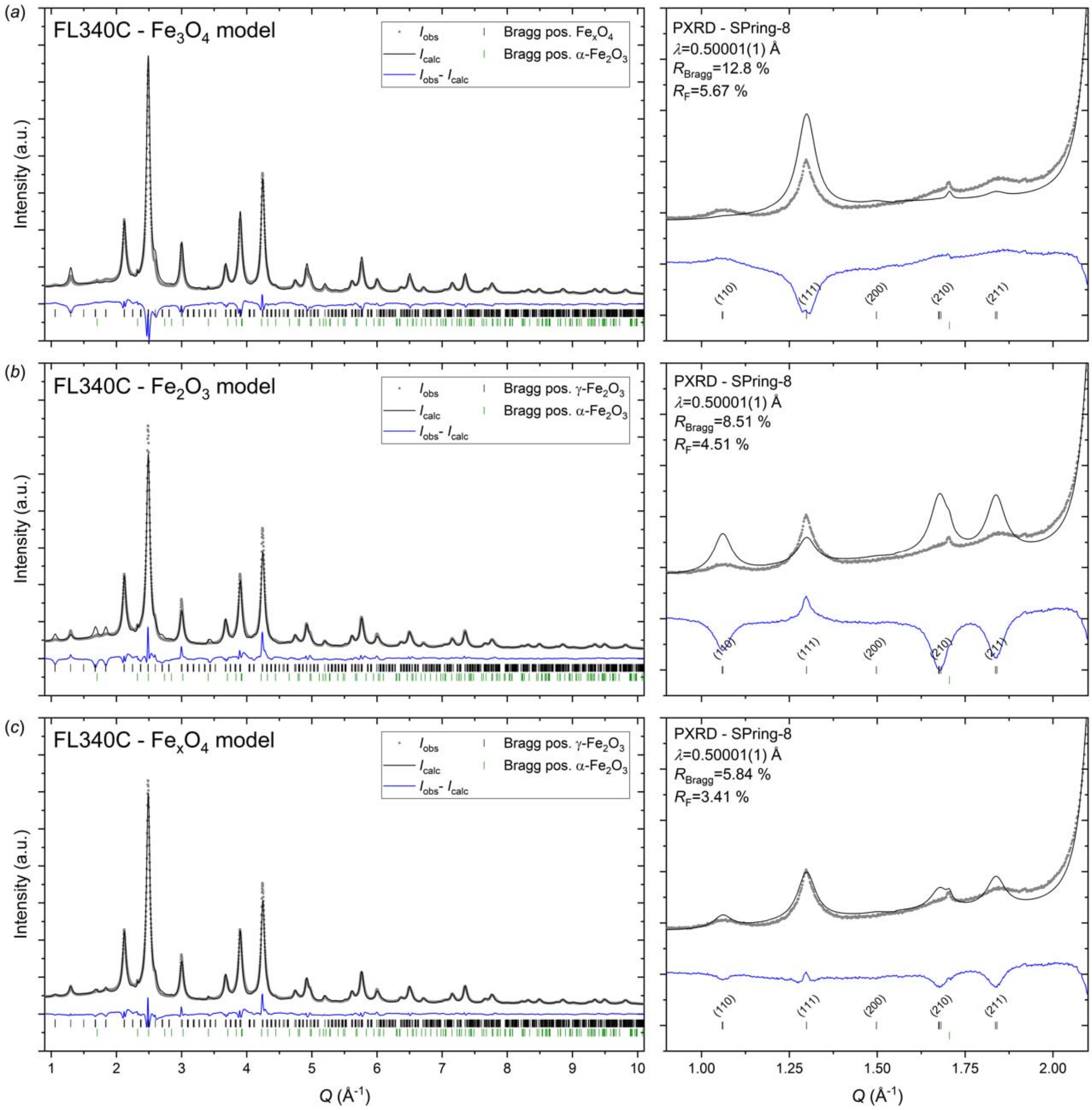

Figure S2 High-resolution synchrotron PXRD data collected on the FL340C sample and corresponding Rietveld refinements in (a) space group $P4_32_12$ using magnetite (Fe_3O_4) stoichiometry, *i.e.* site occupation fraction on Fe4*a* site fixed to 1, (b) maghemite (γ-Fe_2O_3) stoichiometry, *i.e.* site occupation fraction on Fe4*a* site fixed to 0.33 and (c) intermediate (Fe_xO_4) non-stoichiometric, *i.e.* site occupation fraction on Fe4*a* site refined freely. The refinements clearly illustrate the superiority of the non-stoichiometric model, which is evident from the improvement in *R*-factors and the improved description of the superstructure peaks illustrated by the magnifications of the given 2θ-regions on the right.

S3. PXRD – FL390C models

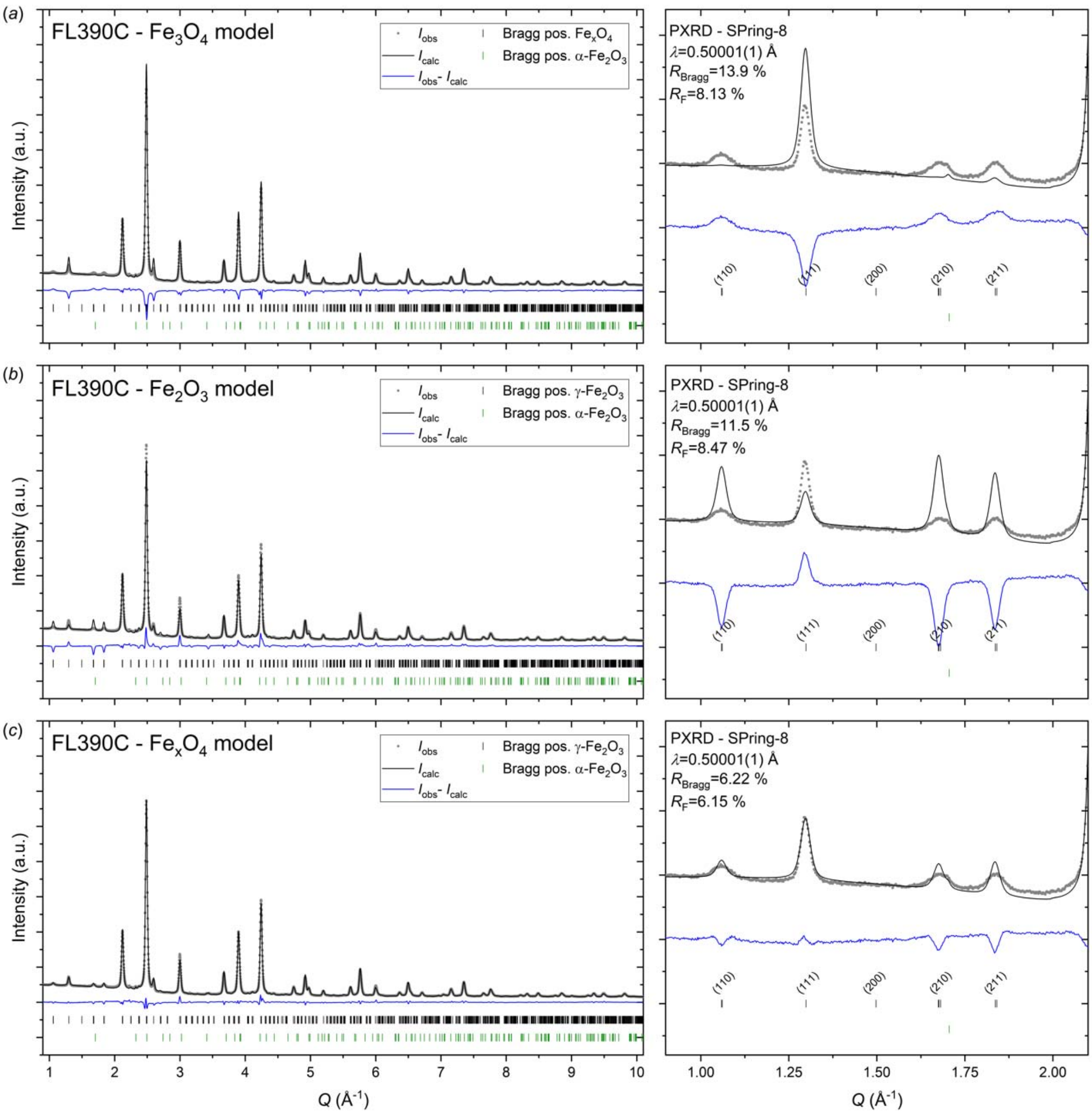

Figure S3 High-resolution synchrotron PXRD data collected on the FL390C sample and corresponding Rietveld refinements in (a) space group $P4_32_12$ using magnetite (Fe_3O_4) stoichiometry, *i.e.* site occupation fraction on Fe4*a* site fixed to 1, (b) maghemite (γ-Fe_2O_3) stoichiometry, *i.e.* site occupation fraction on Fe4*a* site fixed to 0.33 and (c) intermediate (Fe_xO_4) non-stoichiometric, *i.e.* site occupation fraction on Fe4*a* site refined freely. The refinements clearly illustrate the superiority of the non-stoichiometric model, which is evident from the improvement in *R*-factors and the improved description of the superstructure peaks illustrated by the magnifications of the given 2θ-regions on the right.

S4. PXRD – FL440C models

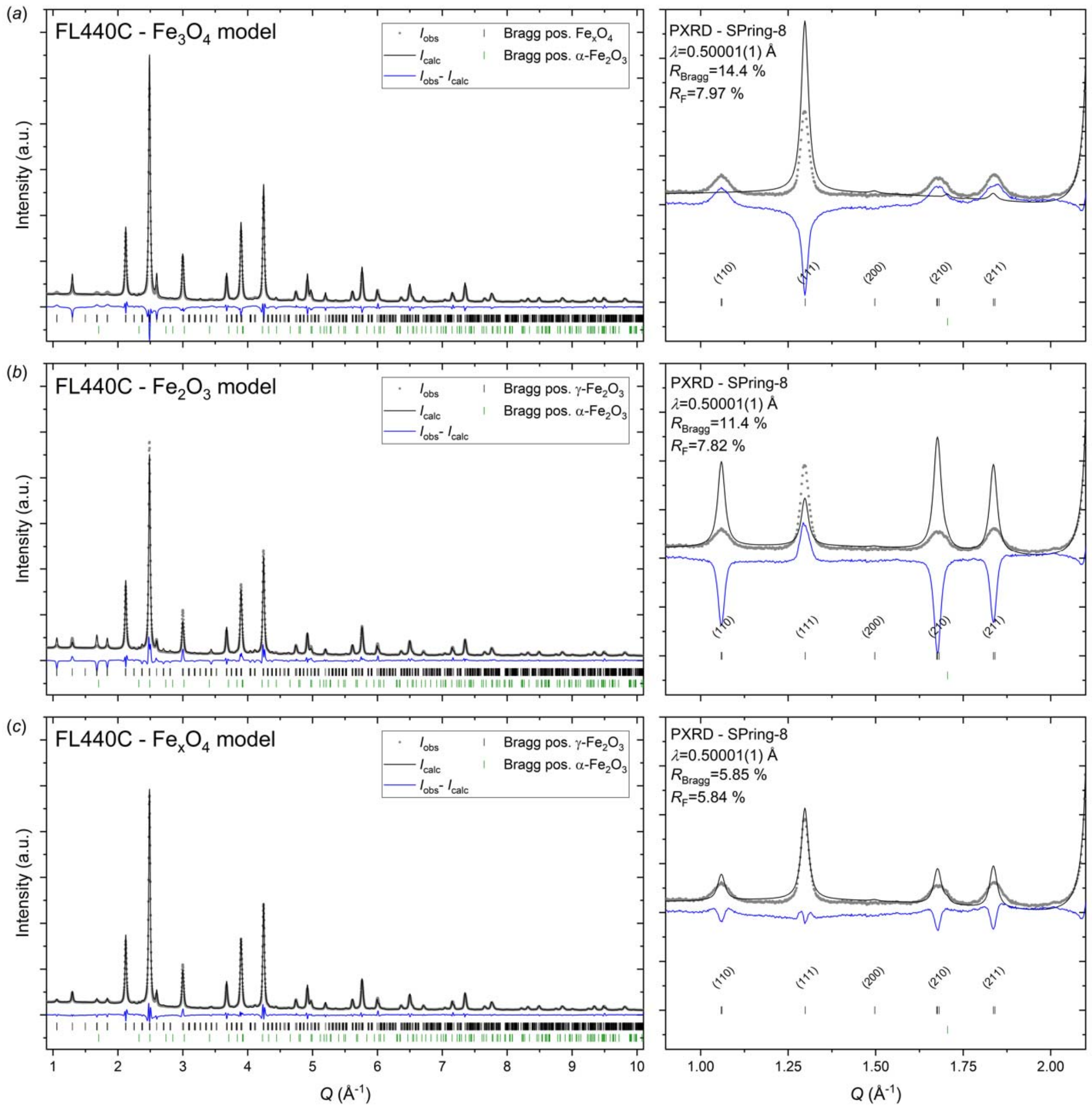

Figure S4 High-resolution synchrotron PXRD data collected on the FL440C sample and corresponding Rietveld refinements in (a) space group $P4_32_12$ using magnetite (Fe_3O_4) stoichiometry, *i.e.* site occupation fraction on Fe4*a* site fixed to 1, (b) maghemite (γ-Fe_2O_3) stoichiometry, *i.e.* site occupation fraction on Fe4*a* site fixed to 0.33 and (c) intermediate (Fe_xO_4) non-stoichiometric, *i.e.* site occupation fraction on Fe4*a* site refined freely. The refinements clearly illustrate the superiority of the non-stoichiometric model, which is evident from the improvement in *R*-factors and the improved description of the superstructure peaks illustrated by the magnifications of the given 2θ-regions on the right.

S5. Microstructural model – Considerations regarding refinement of microstrain

Attempts at implementing a microstrain contribution to the peak profile refinements were unsuccessful. The refined parameters (Gaussian and/or Lorentzian) tend to zero causing either non-converging fits or unphysical negative peak widths. Refining the oxygen B-values (previously fixed to 0.5) allows physically meaningful strain values to be refined, however, at the cost of unphysical negative oxygen displacement factors. Ultimately, it is thus concluded that the effect of any microstrain on the observed diffraction peak profiles are negligible given the very minor difference in cell parameters, *i.e.* a(γ-Fe_2O_3)=8.33 Å and a(Fe_3O_4)=8.396 Å at ambient conditions, between the end-member structures and the relatively large/dominating Scherrer broadening due to the fine crystallite size. In any case, the effect, if any, of isotropic microstrain will not influence the obtained atomic structure or trend in extracted crystallite size.

S6. FL340C – Data, Rietveld fits and refined model

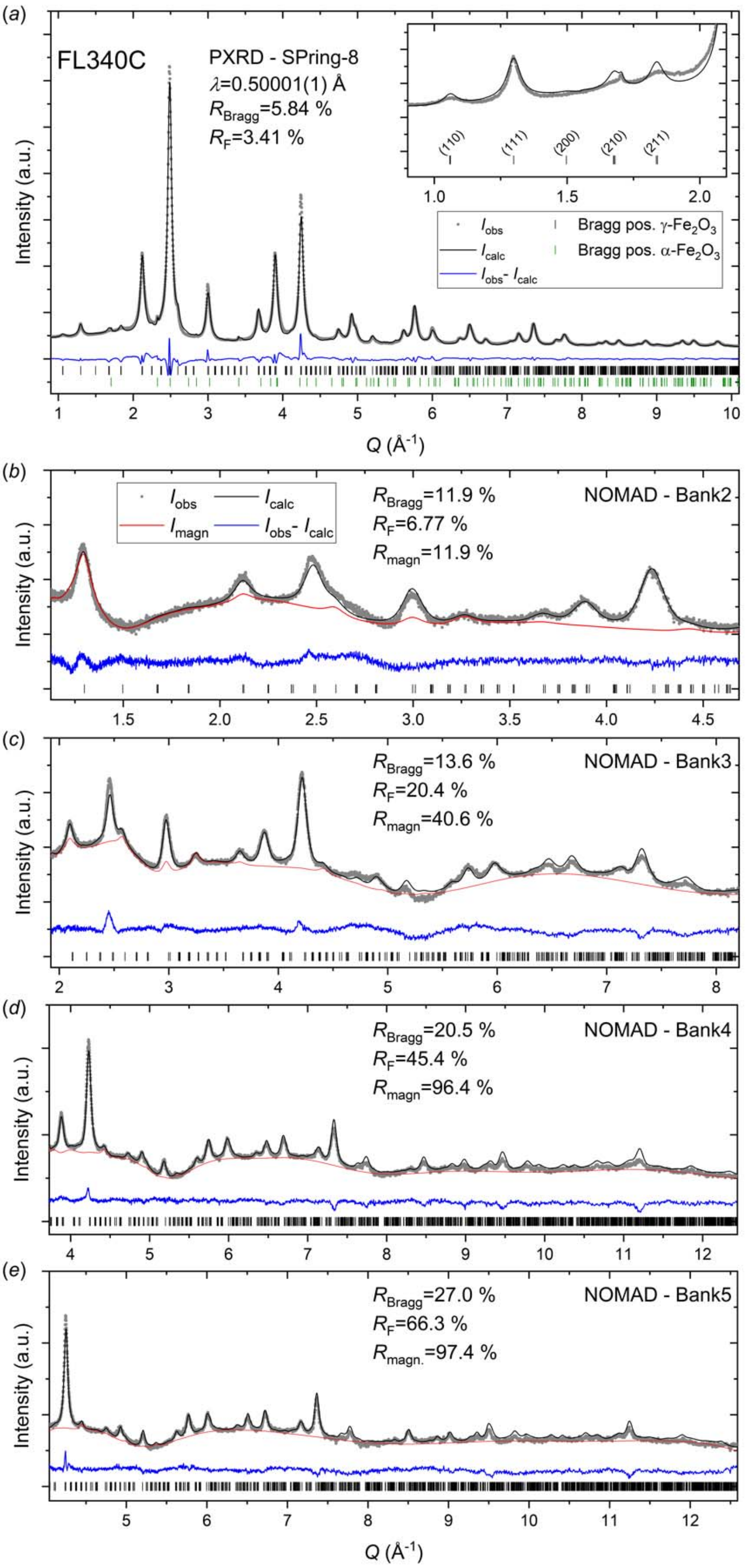

Figure S5 Joint Rietveld refinement of a constrained structural model in space group $P4_32_12$ to (a) high-resolution synchrotron PXRD data and (b-e) neutron time-of-flight datasets from the FL340C sample.

Table S1 The atomic structural model in tetragonal space group $P4_32_12$ (as reported by Greaves,(Greaves, 1983)) employed in the refinement of the FL340C sample.

FL340C (Fe_xO_4)

Space group: $P4_32_12$ (No. 96)

a=b=8.3838(8) Å, c=8.3452(10) Å, α=β=γ=90°

$B_{iso,overall}$=0.20(1) Å^2

Atom	Site	*x*	*y*	*z*	*Sof*
Fe1	8*b*	0.744(3)	0.995(3)	0.119(2)	1
Fe2	4*a*	0.617(1)	x	0	1
Fe3	8*b*	0.364(1)	0.869(2)	-0.013(1)	1
Fe4	4*a*	0.132(3)	x	0	0.583(8)
O1	8*b*	0.614(8)	0.863(10)	-0.013(8)	1
O2	8*b*	0.119(10)	0.377(11)	-0.007(9)	1
O3	8*b*	0.130(9)	0.874(11)	0.006(8)	1
O4	8*b*	0.382(9)	0.627(8)	-0.003(7)	1

Notes: The numbers in parentheses indicate the errors on the last significant digit of the refined parameters. The secondary hematite phase (space group *R*-3*c*) was found to constitute 0.9(5) wt%.

S7. FL390C – Data, Rietveld fits and refined model

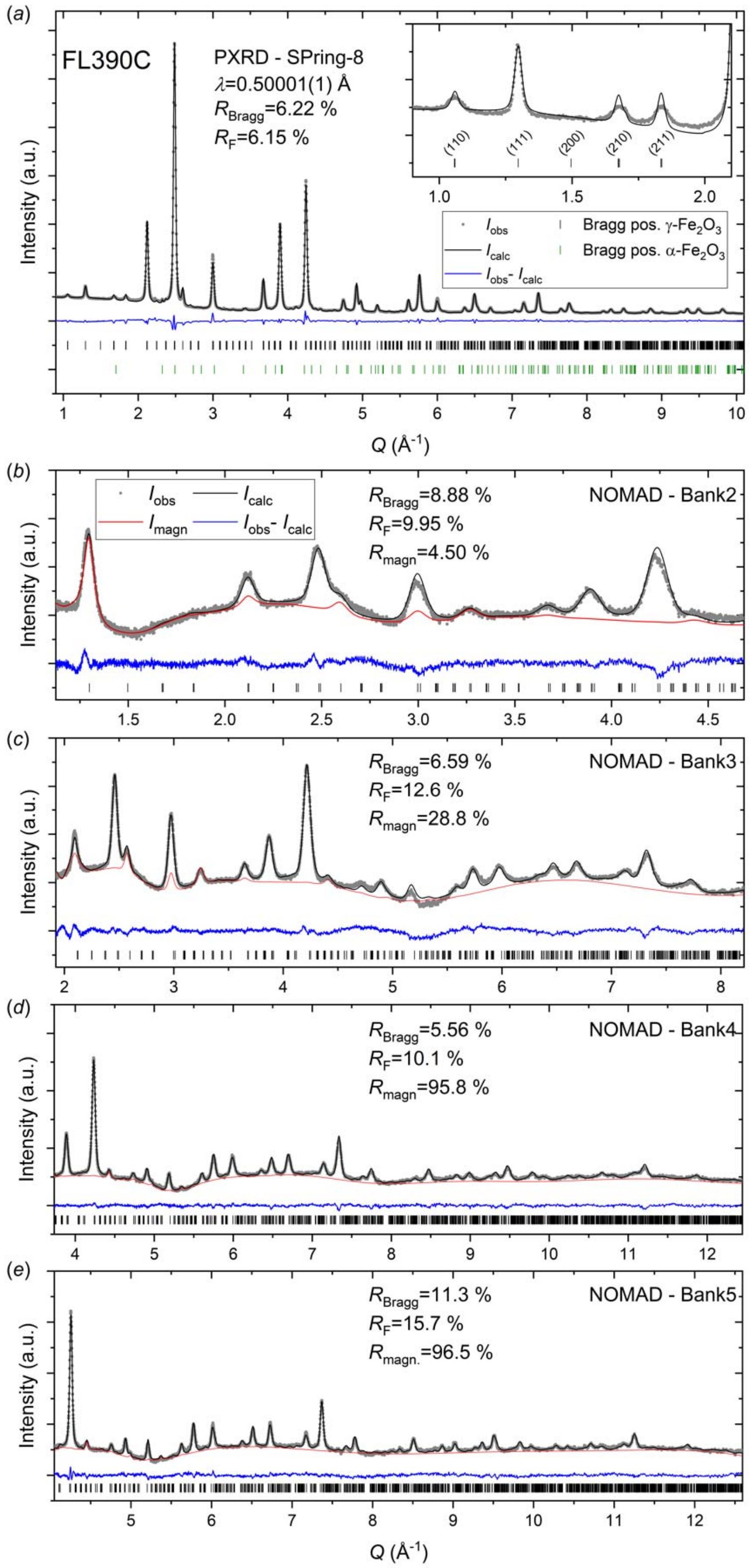

Figure S6 Joint Rietveld refinement of a constrained structural model in space group $P4_32_12$ to (a) high-resolution synchrotron PXRD data and (b-e) neutron time-of-flight datasets from the FL390C sample.

Table S2 The atomic structural model in tetragonal space group $P4_32_12$ (as reported by Greaves,(Greaves, 1983) employed in the refinement of the FL390C sample.

**FL390C (Fe_xO_4)**					
Space group: $P4_32_12$ (No. 96)					
a=b=8.3784(2) Å, c=8.3458(4) Å, α=β=γ=90°					
$B_{iso,overall}$=0.38(1) Å^2					
Atom	Site	*x*	*y*	*z*	*Sof*
Fe1	8*b*	0.745(1)	0.996(2)	0.123(2)	1
Fe2	4*a*	0.620(1)	x	0	1
Fe3	8*b*	0.365(1)	0.871(2)	-0.010(1)	1
Fe4	4*a*	0.126(3)	x	0	0.604(8)
O1	8*b*	0.614(4)	0.873(4)	-0.014(3)	1
O2	8*b*	0.118(3)	0.363(3)	-0.016(3)	1
O3	8*b*	0.112(3)	0.870(3)	0.004(3)	1
O4	8*b*	0.387(3)	0.635(3)	0.008(2)	1

Notes: The numbers in parentheses indicate the errors on the last significant digit of the refined parameters. The secondary hematite phase (space group *R*-3*c*) was found to constitute 0.9(6) wt%.

S8. FL440C – Data, Rietveld fits and refined model

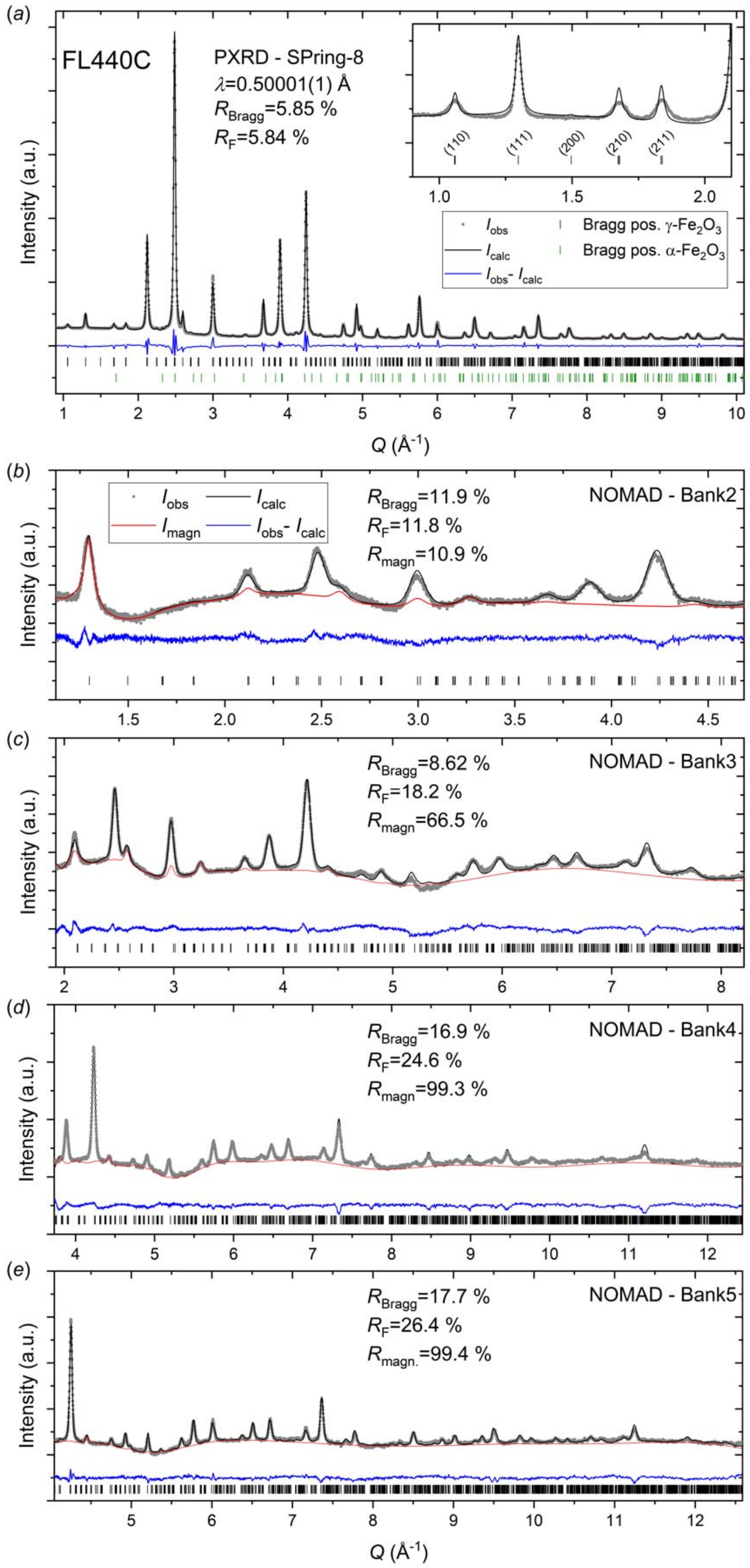

Figure S7 Joint Rietveld refinement of a constrained structural model in space group $P4_32_12$ to (a) high-resolution synchrotron PXRD data and (b-e) neutron time-of-flight datasets from the FL440C sample.

Table S3 The atomic structural model in tetragonal space group $P4_32_12$ (as reported by Greaves,(Greaves, 1983)) employed in the refinement of the FL440C sample.

FL440C (Fe_xO_4)

Space group: $P4_32_12$ (No. 96)

a=b=8.3846(4) Å, c=8.3461(4) Å, α=β=γ=90°

$B_{iso,overall}$=0.26(1) Å^2

Atom	Site	*x*	*y*	*z*	*Sof*
Fe1	8*b*	0.746(2)	0.996(2)	0.121(1)	1
Fe2	4*a*	0.621(1)	x	0	1
Fe3	8*b*	0.367(1)	0.869(1)	-0.010(1)	1
Fe4	4*a*	0.131(2)	x	0	0.621(7)
O1	8*b*	0.615(4)	0.865(5)	-0.012(4)	1
O2	8*b*	0.115(5)	0.380(6)	-0.007(4)	1
O3	8*b*	0.132(6)	0.876(6)	0.008(4)	1
O4	8*b*	0.383(5)	0.627(5)	0.003(3)	1

Notes: The numbers in parentheses indicate the errors on the last significant digit of the refined parameters. The secondary hematite phase (space group *R*-3*c*) was found to constitute 0.7(5) wt%.

S9. PXRD – FL340C superstructure peak profile analysis

The peak profile analysis in the refinement of the whole pattern yielded a mean crystallite diameter of the FL340C sample of 8.71(8) nm. However, a moderate discrepancy between the fitted model and the superstructure reflections is observed. Applying Scherrer analysis to the (210) and (211) superstructure peaks alone indicates a vacancy-ordered domain size of 8(1) nm, while single-peak fitting of the (220) reflection indicates a spinel domain size of 9.4(3) nm.

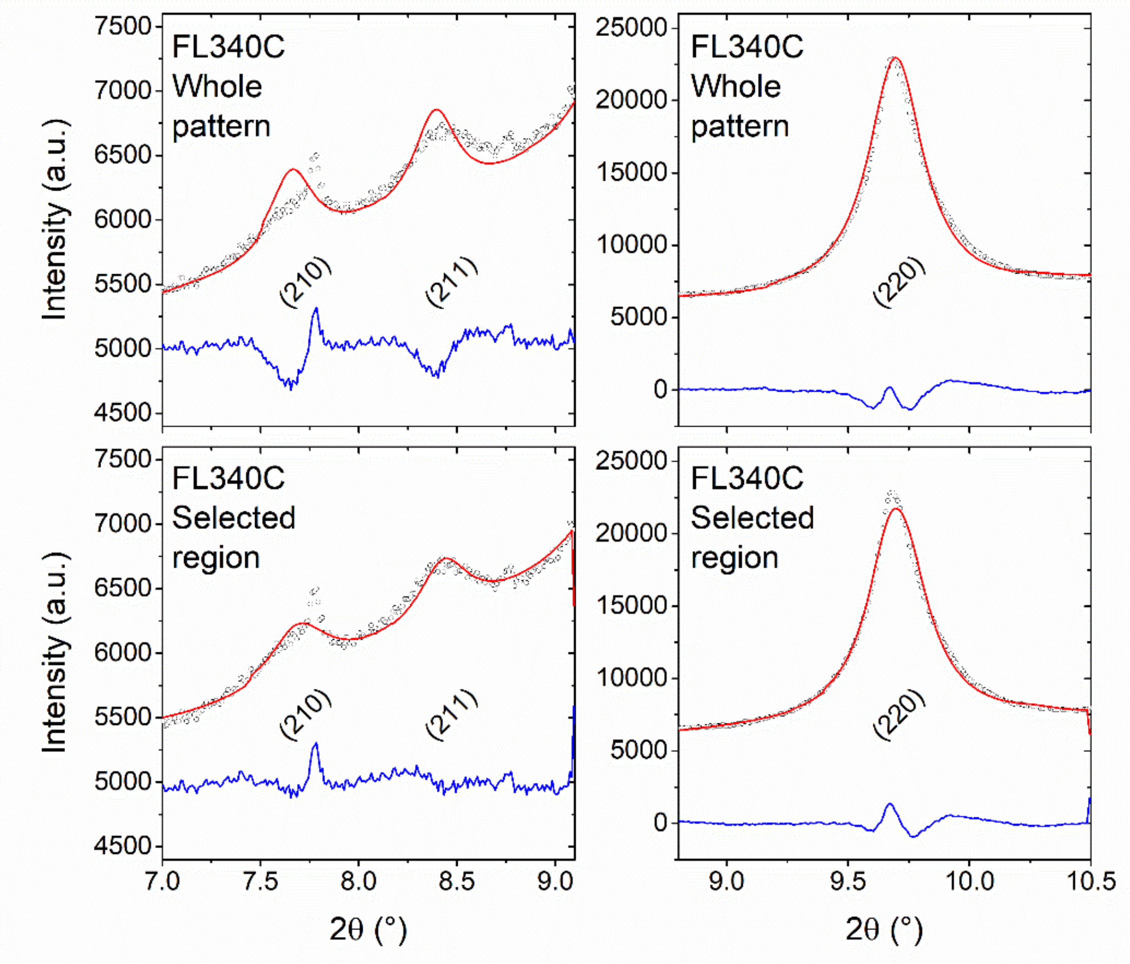

Figure S8 Magnification of the 2θ-region containing the (210) and (211) families of superstructure reflections (left) and of the region containing the (220) spinel reflection in the synchrotron PXRD data (black) of FL340C. The fits (red) obtained when refining the full pattern are illustrated in the top while fits obtained when refining only the shown 2θ-region containing the give reflections are shown in the bottom.

S10. PXRD – FL390C superstructure peak profile analysis

The peak profile analysis in the refinement of the whole pattern yielded a mean crystallite diameter of the FL390C sample of 15.6(2) nm. However, a moderate discrepancy between the fitted model and the superstructure reflections is observed. Applying Scherrer analysis to the (210) and (211) superstructure peaks alone indicates a vacancy-ordered domain size of 10(2) nm, while single-peak fitting of the (220) reflection indicates a spinel domain size of 18.6(10) nm.

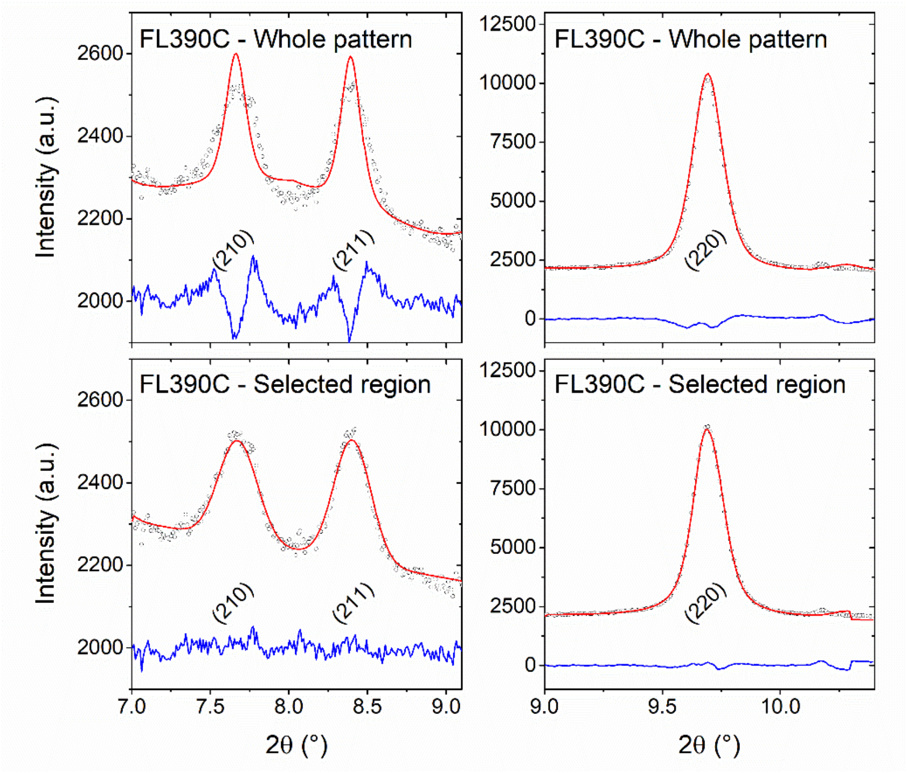

Figure S9 Magnification of the 2θ-region containing the (210) and (211) families of superstructure reflections (left) and of the region containing the (220) spinel reflection in the synchrotron PXRD data (black) of FL390C. The fits (red) obtained when refining the full pattern are illustrated in the top while fits obtained when refining only the shown 2θ-region containing the give reflections are shown in the bottom.

S11. PXRD – FL440C superstructure peak profile analysis

The peak profile analysis in the refinement of the whole pattern yielded a mean crystallite diameter of the FL440C sample of 17.0(2) nm. However, a moderate discrepancy between the fitted model and the superstructure reflections is observed. Applying Scherrer analysis to the (210) and (211) superstructure peaks alone indicates a vacancy-ordered domain size of 10(3) nm, while single-peak fitting of the (220) reflection indicates a spinel domain size of 20.5(6) nm.

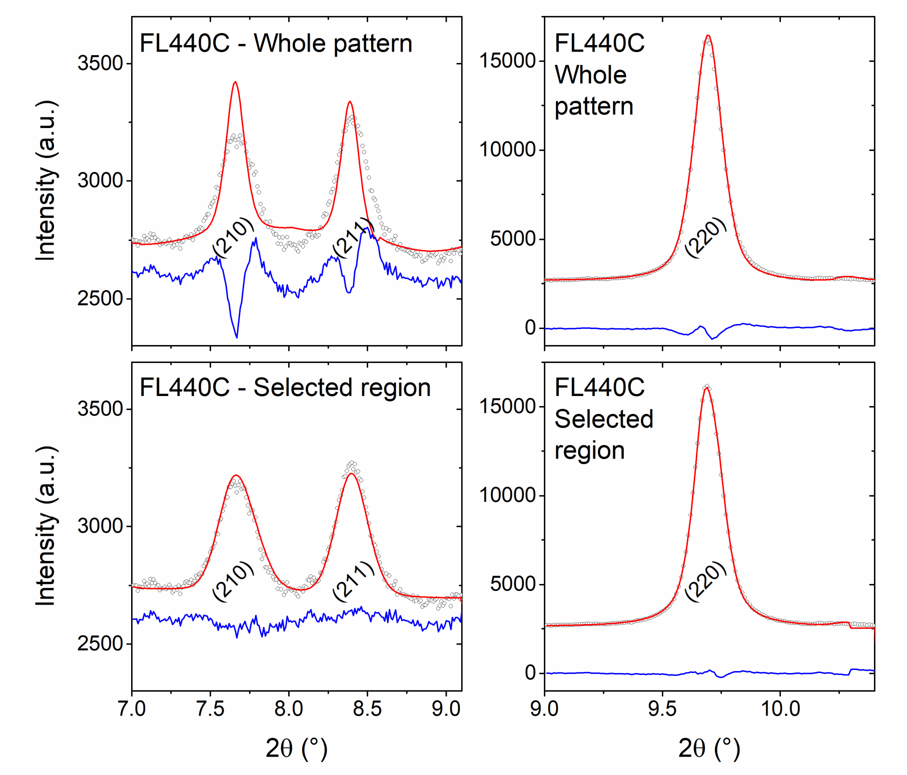

Figure S10 Magnification of the 2θ-region containing the (210) and (211) families of superstructure reflections (left) and of the region containing the (220) spinel reflection in the synchrotron PXRD data (black) of FL440C. The fits (red) obtained when refining the full pattern are illustrated in the top while fits obtained when refining only the shown 2θ-region containing the give reflections are shown in the bottom.

S12. TEM – FL340C additional images

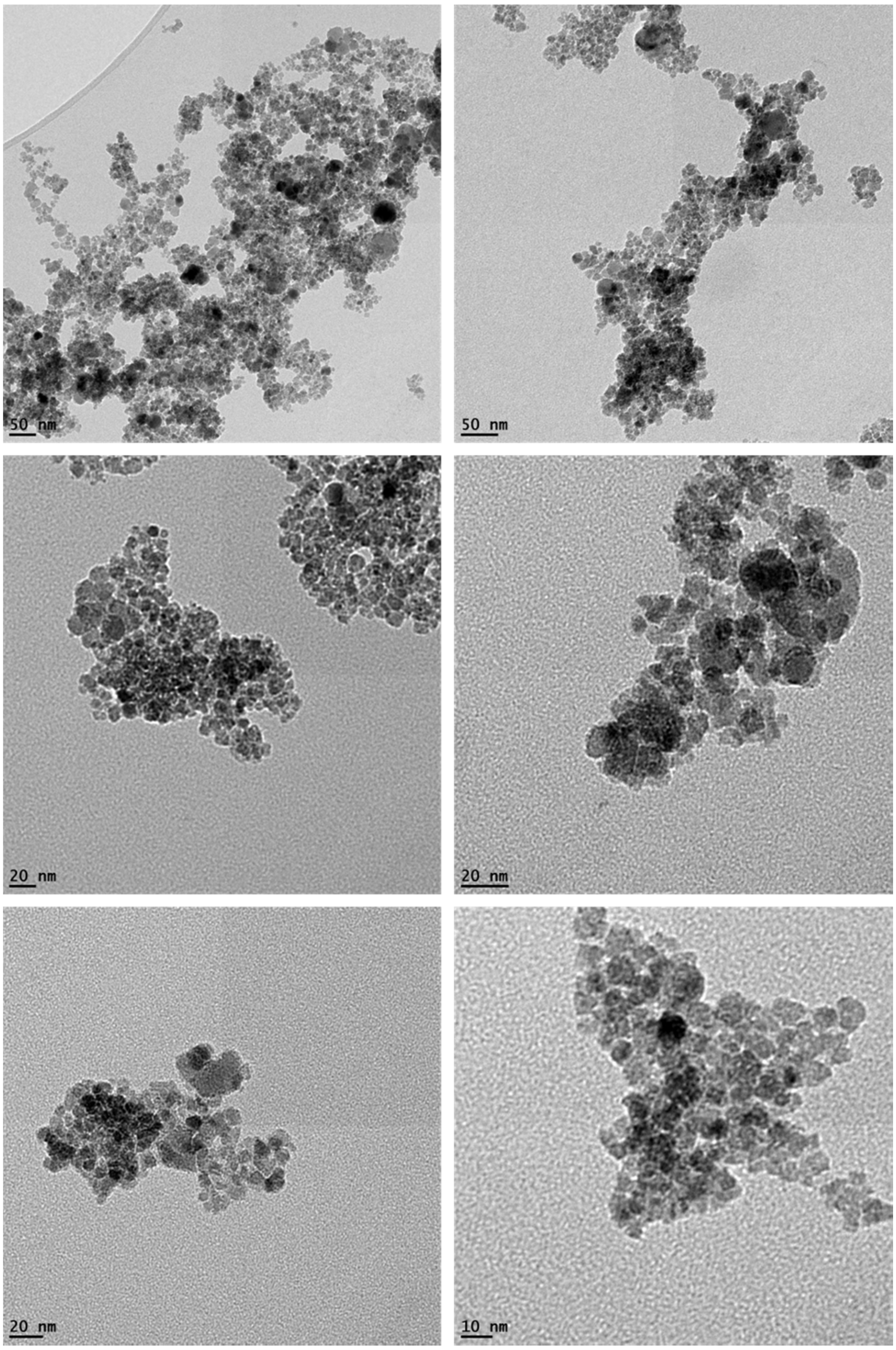

Figure S11 TEM images of the nanoparticles in the FL340C sample. Size analysis based on manual measurement of the diameter of 212 particles.

S13. TEM – FL390C additional images

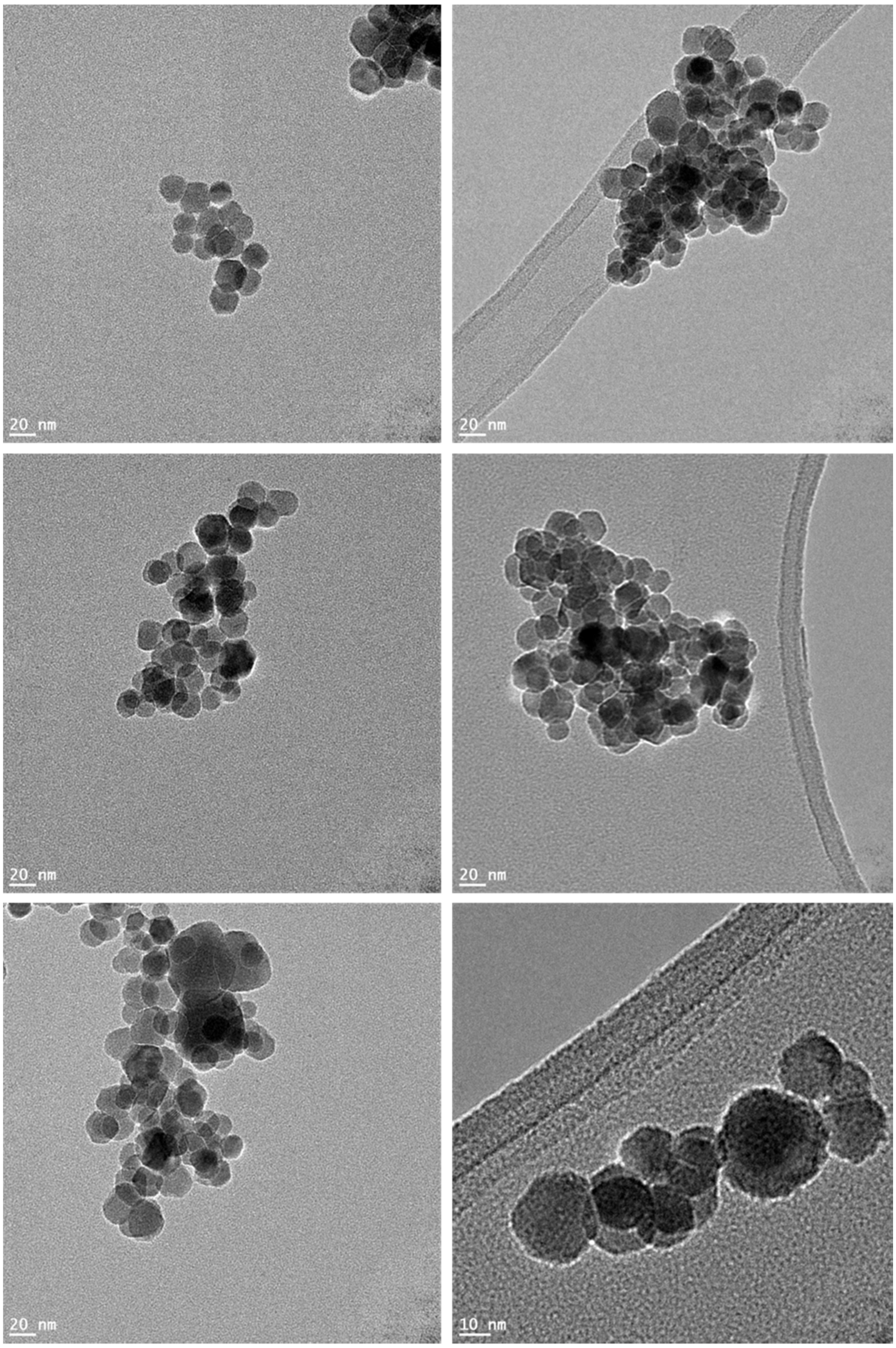

Figure S12 TEM images of the nanoparticles in the FL390C sample. Size analysis based on manual measurement of the diameter of 196 particles.

S14. TEM – FL440C additional images

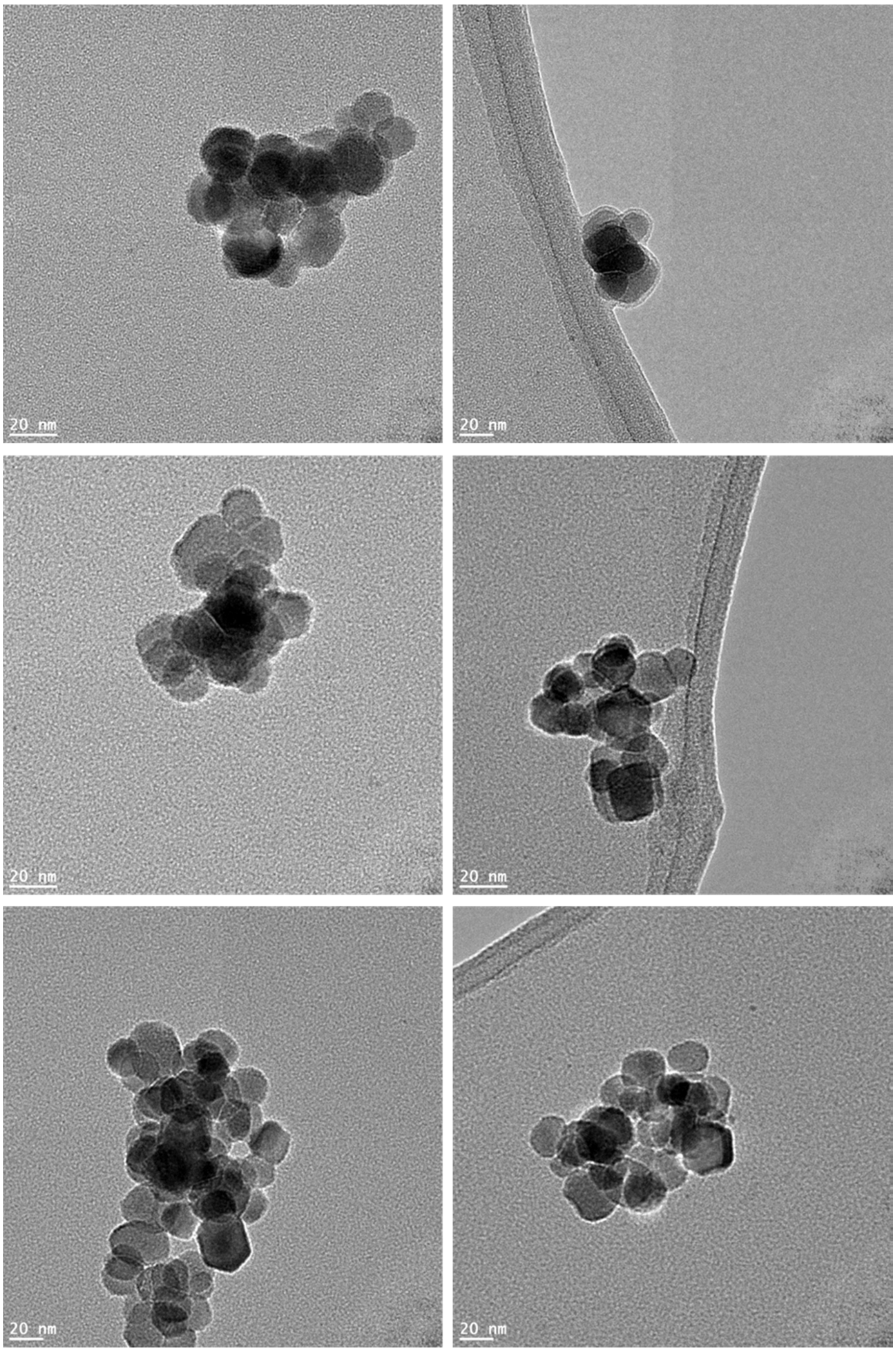

Figure S13 TEM images of the nanoparticles in the FL440C sample. Size analysis based on manual measurement of the diameter of 99 particles.

S15. STEM-EDS – FL440C elemental maps

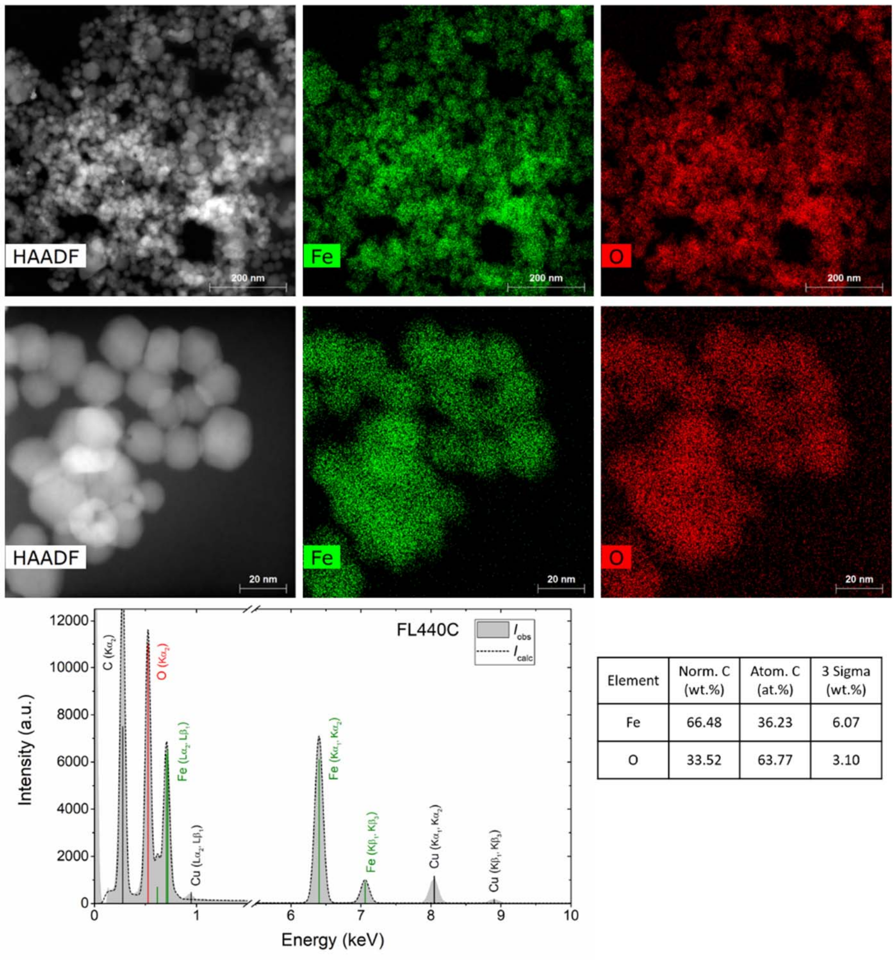

Element	Norm. C (wt.%)	Atom. C (at.%)	3 Sigma (wt.%)
Fe	66.48	36.23	6.07
O	33.52	63.77	3.10

Figure S14 STEM-HAADF images and elemental maps illustrating the distribution of Fe and O in the FL440C nanoparticles. The corresponding energy-dispersive X-ray spectrum including quantitative elemental analysis is shown in the bottom.

S16. STEM-EDS – FL440C line scans

Fig. S15(*a*) shows a STEM-HAADF image of the FL440C sample and the corresponding elemental STEM-EDS maps of Fe and O. The maps indicate elemental homogeneity in the FL440C nanoparticles. Quantitative analysis of the EDS spectrum from the mapped area was performed, however, an accurate determination of the exact Fe_xO_4 stoichiometry could not be obtained, due to the oxygen background signal, which cannot be discerned from the oxygen in the sample and thus leads to an underestimation Fe/O ratio (see Supporting Information). The background signal is likely to originate from residual solvent (water or ethanol) adsorbed onto the TEM grid. In addition, STEM-EDS line scans across single particles were carried out in order to test whether the prepared spinel iron oxide nanoparticles consist of a single homogeneous Fe_xO_4 phase, a core-shell nanostructure or comprise a gradient structure with a Fe-rich centre and an oxidized surface. Line scans and corresponding elemental counts of two different nanoparticles are shown in Fig. S15 (*d*-*e*) and the relative changes in Fe/O ratio across the particles are shown in Fig. S15 (*f*-*g*).

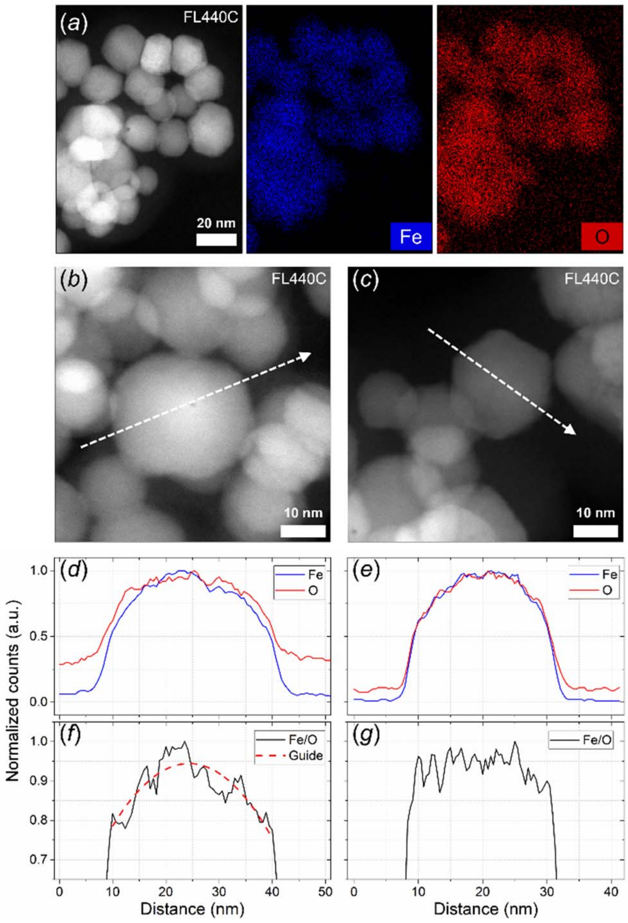

Figure S15 STEM-EDS elemental analysis of particles in the FL440C sample. (*a*) HAADF-STEM image and corresponding elemental maps of Fe and O in the region. (*b*-*c*) HAADF-STEM images with arrows indicating the location of conducted 1D line scans across single particles. (*d*-*e*) Corresponding normalized Fe and O counts as function of position. (*f*-*g*) Normalized Fe/O ratio as

function of position. A second-degree polynomial fit has been added to (*f*) as a guide to the eye. Additional STEM-EDS line scans may be found in the Supporting Information.

The STEM-EDS line scans do not indicate the presence of an ideal core-shell like nanostructure but do show minor hints of an increased Fe/O-ratio towards the centre of the particles (in particular that of the largest particle of ≈30 nm, see Fig. 5 (*f*)). Considering the highest Fe/O-ratio present in the samples to come from Fe_3O_4, this would give a normalized Fe/O value of 1 for the Fe_3O_4 and 0.88 for the γ-Fe_2O_3 stoichiometries, respectively. Given the small difference between these two values and the relatively large variations in the Fe/O line scan curves, the STEM-EDS data alone cannot be considered conclusive evidence of a particular nanostructural model.

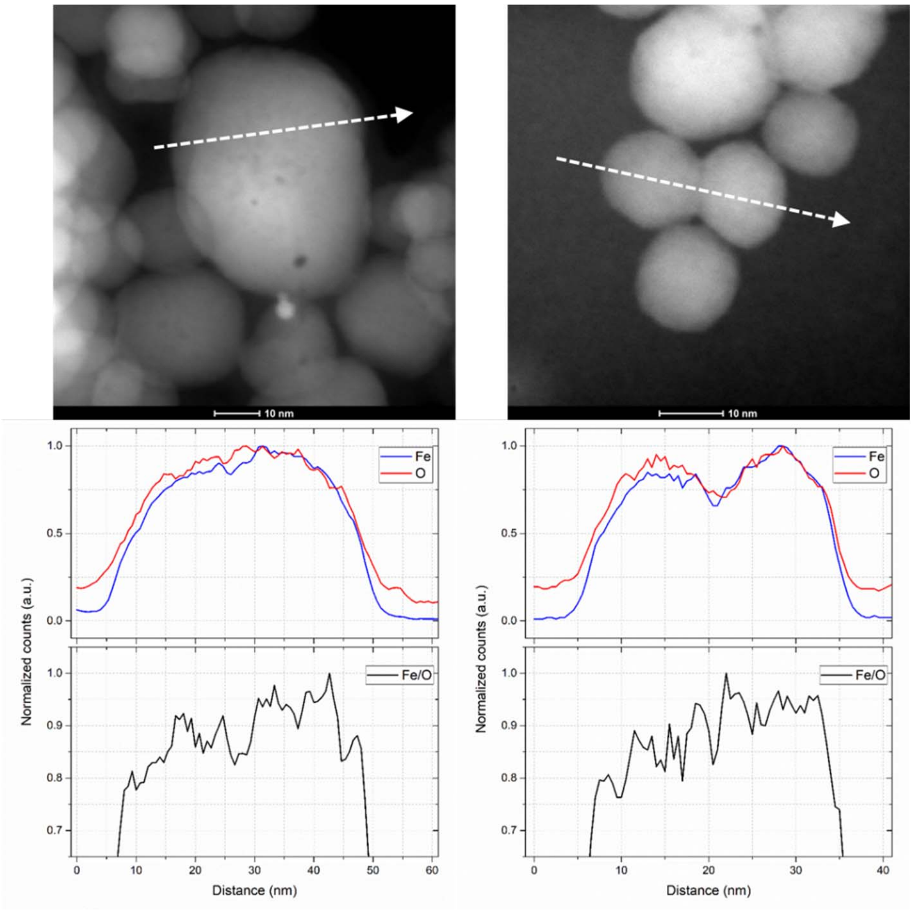

Figure S16 Additional STEM-EDS line scans showing the elemental distribution across nanoparticles in the FL440C sample.

S17. X-ray TS-PDF refinement

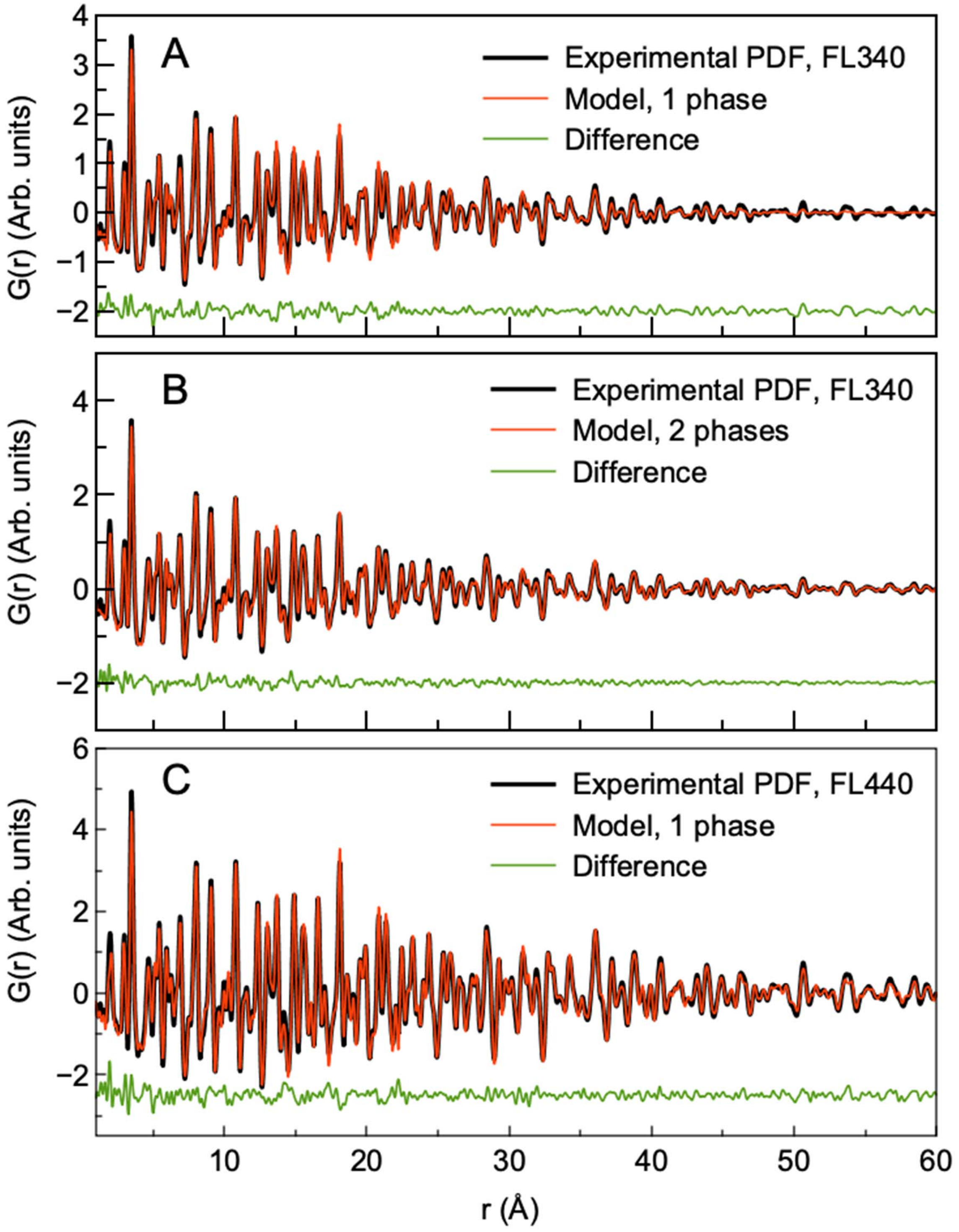

Figure S17 Fits to the X-ray PDFs: a) Sample FL340, modelled in range 1-60 Å using one maghemite phase and b) Sample FL340, modelled in range 1-60 Å using two maghemite phases. c) Sample FL440, modelled in range 1-60 Å using one maghemite phase.

Table S4 Summary of results from the PDF analysis of the synchrotron X-ray total scattering data, fitted in the range 1-20 Å. *The refined sp-diameter from a short-range fit does not reflect the actual crystallite size.

	**FL340C**	**FL440C**
**Data**		
Instrument	ID11, ESRF	ID11, ESRF
Wavelength, λ (Å)	0.1897	0.1897
Termination, q_{max} (Å^{-1})	23.5	23.5
Dampening, q_{damp} (Å^{-1})	0.02895	0.02895
Data range in r (Å)	1-20	1-20
Step dr (Å)	0.01	0.01
**Fit quality**		
R_w	10.6%	10.2%
**Crystal/local structure**		
Space group	$P4_32_12$	$P4_32_12$
Stoichiometry, Fe_xO_4, x	2.70	2.85
Scale factor	0.33	0.41
a (Å)	8.402	8.413
c (Å)	8.323	8.352
Delta2	3.33	3.44
sp-diameter* (Å)	50	100

Table S5 The atomic structural model in tetragonal space group $P4_32_12$ obtained from the PDF analysis of the FL340C X-ray total scattering data.

Site	Wyckoff pos.	*x*	*y*	*z*	u_{iso}, phase 1	Site occupation fraction
**Fe(1)**	8*b*	0.745	0.996	0.121	0.005	1
**Fe(2)**	4*a*	0.623	0.623	0	0.005	1
**Fe(3)**	8*b*	0.369	0.868	0.989	0.005	1
**Fe(4)**	4*a*	0.130	0.130	0	0.005	0.39
**O(1)**	8*b*	0.620	0.872	0.982	0.001	1
**O(2)**	8*b*	0.092	0.402	0.991	0.001	1
**O(3)**	8*b*	0.132	0.881	0.007	0.001	1
**O(4)**	8*b*	0.365	0.632	0.998	0.001	1

Table S6 The atomic structural model in tetragonal space group $P4_32_12$ obtained from the PDF analysis of the FL440C X-ray total scattering data.

Site	Wyckoff pos.	*x*	*y*	*z*	u_{iso}, phase 1	Site occupation fraction
**Fe(1)**	8*b*	0.749	0.000	0.118	0.004	1
**Fe(2)**	4*a*	0.623	0.623	0	0.006	1
**Fe(3)**	8*b*	0.377	0.879	0.996	0.006	1
**Fe(4)**	4*a*	0.128	0.128	0	0.006	0.69
**O(1)**	8*b*	0.559	0.860	0.976	0.004	1
**O(2)**	8*b*	0.109	0.376	0.011	0.004	1
**O(3)**	8*b*	0.138	0.881	0.002	0.004	1
**O(4)**	8*b*	0.383	0.618	0.990	0.004	1

Table S7 Summary of results from the PDF analysis of the synchrotron X-ray total scattering data, fitted in the range 1-60 Å. Sample FL340 was described with two Fe_2O_3 phases with different crystallite sizes to reflect the large distribution of crystallite sizes present in the sample.

	**FL340C**	**FL440C**
**Data**		
Instrument	ID11, ESRF	ID11, ESRF
Wavelength, λ (Å)	0.1897	0.1897
Termination, q_{max} (Å^{-1})	23.5	23.5
Dampening, q_{damp} (Å^{-1})	0.02895	0.02895
Data range in r (Å)	1-30	1-30
Step dr (Å)	0.01	0.01
**Fit quality**		
R_w	12.4%	14.0%
**Crystal/local structure**		
Space group, phase 1 and 2	$P4_32_12$	$P4_32_12$
Stoichiometry, Fe_xO_4, x phase 1 and 2	2.72	2.85
Scale factor, phase 1	0.18	0.38
Weight percent, phase 1	52%	-
a (Å), phase 1	8.402	8.411
c (Å), phase 1	8.341	8.353
Delta2, phase 1	3.13	3.26
sp-diameter, phase 1 (Å)	110	134
Scale factor, phase 2	0.16	-
Weight percent, phase 2	48%	-
a (Å), phase 2	8.42	-
c (Å), phase 2	8.36	-
Delta2, phase 2	3.13	-
sp-diameter, phase 2	26	-

S18. Mössbauer analysis

The room temperature Mössbauer spectra of the three nanocrystalline spinel iron oxide powders are shown in Fig. S18. The dominating component in the Mössbauer spectra of FL390 and FL440C is a sextet-like feature due to Fe^{3+}. Based on the negligible quadrupole interaction and isomer-shift δ_{RT} = 0.335(5) mm/s (relative to metallic iron), it is attributed to particles showing slow superparamagnetic relaxations compared to the timescale of Mössbauer spectroscopy (~10^7 Hz). The spectrum of FL340 the sextet is much smaller and the spectrum is dominated by a quadrupole doublet due to Fe^{3+}. Low temperature measurements show that this component transforms into a sextet like feature, allowing us to conclude that the signal is due to Fe^{3+} showing fast superparamagnetic relaxations. All spectra show an absorption feature at v~-6.5 mm/s. Such a feature is characteristic for the B-line of magnetite, due to mixed valence Fe on octahedral sites. In the final analysis, the spectra were analysed in terms of the three components mentioned above. A model for the spectrum of magnetite was used as the sextet component due to Fe^{3+} on tetrahedral sites in magnetite overlaps the maghemite component. The sample compositions found in the Mössbauer analyses are given in Table 2 and a compilation of the fitted parameters may be found below.

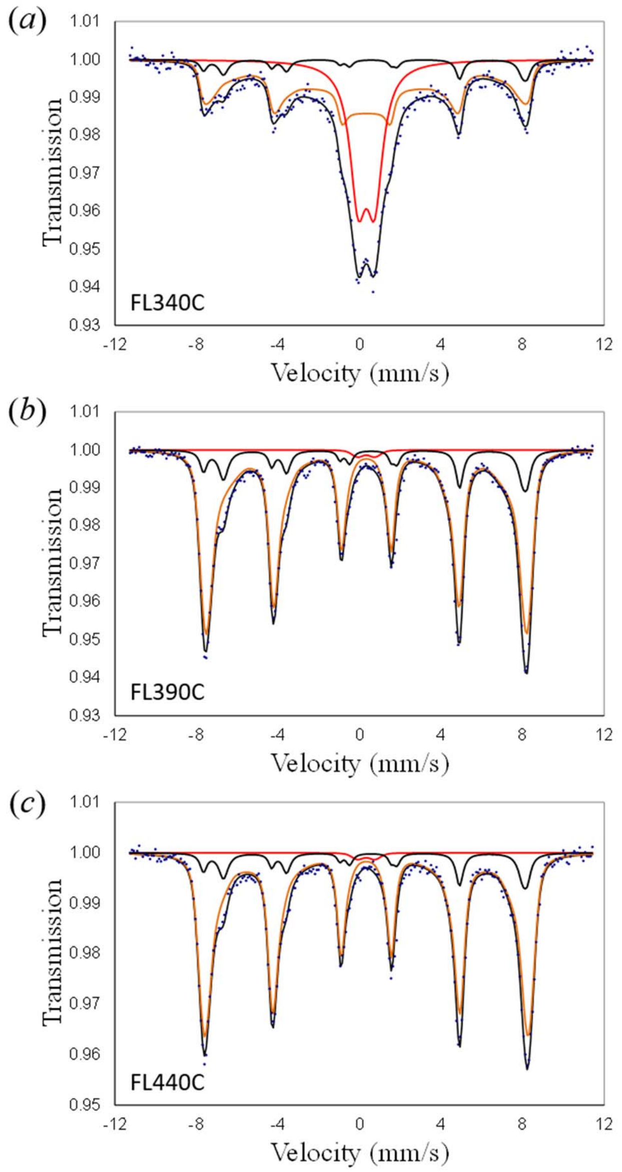

Figure S18 Room temperature Mössbauer spectra of samples synthesized at (*a*) 340 °C, (*b*) 390 °C and (C) 440 °C. The data has been modelled by three components; a superparamagnetic Fe^{3+} doublet (red), a sextet from Fe^{3+} below the superparamagnetic blocking temperature (T_B) (yellow) and a general line-shape of magnetite (black).

S19. Mössbauer – Room temperature spectra analysis parameters

Table S8 Hyperfine parameters and area fractions obtained from the analysis of the spectrum of FL340C. The values in parenthesis represent the error in the last digit. Values with omitted errors were not included as fitting variables.

FL340C – 300 K	MHFD	Fe(III)	Mt-A	Mt-B
B_{peak} (T)	50(2)		49.2	45.5
B_{av} (T)	31(2)			
δ (mm/s)	0.32(2)	0.33(1)	0.29	0.67
ΔE_Q or 2ε (mm/s)	-0.02(3)	0.81(2)	0	0
Γ (mm/s)	0.38(6)	0.96(8)	0.35	0.475
σ (mm/s)		0.00(4)		
Area (%)	56(4)	35(2)	7(2)	

Table S9 Hyperfine parameters and area fractions obtained from the analysis of the spectrum of FL390C. The values in parenthesis represent the error in the last digit. Values with omitted errors were not included as fitting variables.

FL390C – 300 K	MHFD	Fe(III)	Mt-A	Mt-B
B_{peak} (T)	48.9(1)		49.2	45.5
B_{av} (T)	46.2(1)			
δ (mm/s)	0.335(2)	0.33	0.29	0.67
ΔE_Q or 2ε (mm/s)	-0.003(4)	0.84	0	0
Γ (mm/s)	0.38(2)	0.34	0.35	0.475
σ (mm/s)		0.21		
Area (%)	84(2)	1.1(2)	14.3(8)	

Table S10 Hyperfine parameters and area fractions obtained from the analysis of the spectrum of FL440C. The values in parenthesis represent the error in the last digit. Values with omitted errors were not included as fitting variables.

<table>
<tr><th>FL440C – 300 K</th><th>MHFD</th><th>Fe(III)</th><th>Mt-A</th><th>Mt-B</th></tr>
<tr><td>B_{peak} (T)</td><td>49.2(2)</td><td></td><td rowspan="2">49.2</td><td rowspan="2">45.5</td></tr>
<tr><td>B_{av} (T)</td><td>46.6(2)</td><td></td></tr>
<tr><td>δ (mm/s)</td><td>0.335(3)</td><td>0.33</td><td>0.29</td><td>0.67</td></tr>
<tr><td>ΔE_Q or 2ε (mm/s)</td><td>0.003(5)</td><td>0.84</td><td>0</td><td>0</td></tr>
<tr><td>Γ (mm/s)</td><td>0.38(2)</td><td>0.34</td><td>0.35</td><td>0.475</td></tr>
<tr><td>σ (mm/s)</td><td></td><td>0.21</td><td></td><td></td></tr>
<tr><td>Area (%)</td><td>86(3)</td><td>1.1(3)</td><td colspan="2">12.5(10)</td></tr>
</table>

S20. Mössbauer – FL340C low temperature spectra

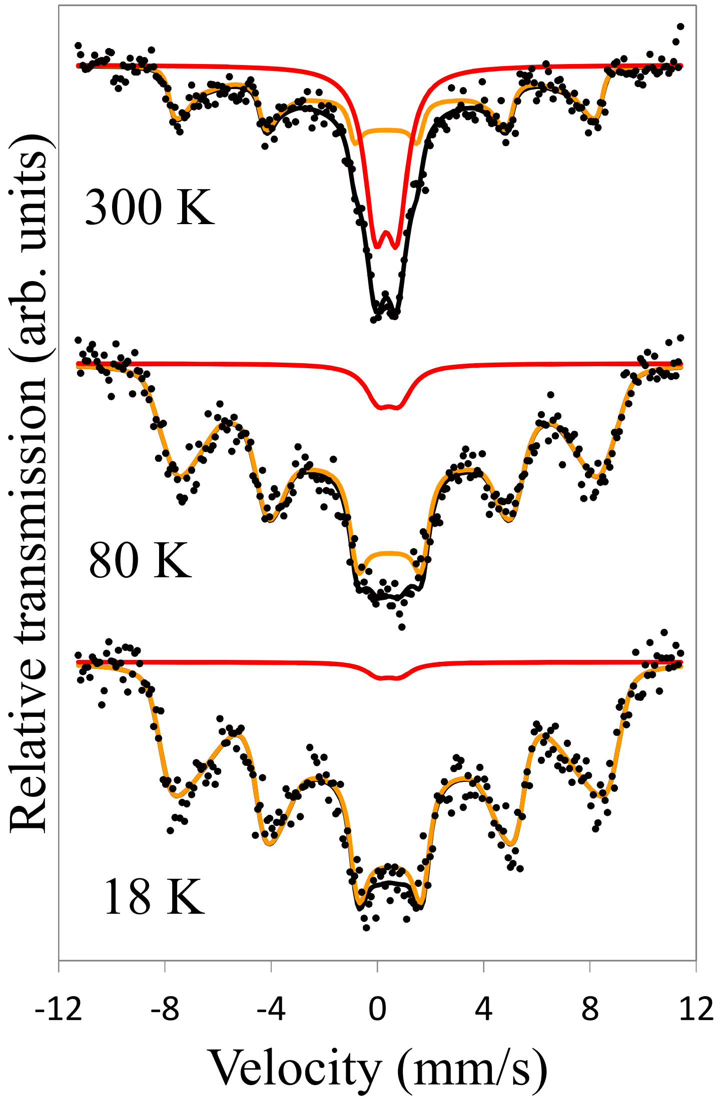

Figure S19 Variable temperature Mössbauer data collected on the FL340C sample. The data has been fitted by two components; a double assigned to Fe^{3+} (red) showing superparamagnetic fluctuations and a sextet distribution assigned to Fe^{3+} (yellow) below the superparamagnetic blocking temperature. The data shows a clear increase in the number of unblocked particles exhibiting superparamagnetic fluctuations as temperature increases.
